# Diversity and Evolutionary History of Iron Metabolism Genes in Diatoms

**DOI:** 10.1371/journal.pone.0129081

**Published:** 2015-06-08

**Authors:** Ryan D. Groussman, Micaela S. Parker, E. Virginia Armbrust

**Affiliations:** School of Oceanography, University of Washington, Seattle, Washington, United States of America; CINVESTAV-IPN, MEXICO

## Abstract

Ferroproteins arose early in Earth’s history, prior to the emergence of oxygenic photosynthesis and the subsequent reduction of bioavailable iron. Today, iron availability limits primary productivity in about 30% of the world’s oceans. Diatoms, responsible for nearly half of oceanic primary production, have evolved molecular strategies for coping with variable iron concentrations. Our understanding of the evolutionary breadth of these strategies has been restricted by the limited number of species for which molecular sequence data is available. To uncover the diversity of strategies marine diatoms employ to meet cellular iron demands, we analyzed 367 newly released marine microbial eukaryotic transcriptomes, which include 47 diatom species. We focused on genes encoding proteins previously identified as having a role in iron management: iron uptake (high-affinity ferric reductase, multi-copper oxidase, and Fe(III) permease); iron storage (ferritin); iron-induced protein substitutions (flavodoxin/ferredoxin, and plastocyanin/cytochrome c6) and defense against reactive oxygen species (superoxide dismutases). Homologs encoding the high-affinity iron uptake system components were detected across the four diatom Classes suggesting an ancient origin for this pathway. Ferritin transcripts were also detected in all Classes, revealing a more widespread utilization of ferritin throughout diatoms than previously recognized. Flavodoxin and plastocyanin transcripts indicate possible alternative redox metal strategies. Predicted localization signals for ferredoxin identify multiple examples of gene transfer from the plastid to the nuclear genome. Transcripts encoding four superoxide dismutase metalloforms were detected, including a putative nickel-coordinating isozyme. Taken together, our results suggest that the majority of iron metabolism genes in diatoms appear to be vertically inherited with functional diversity achieved via possible neofunctionalization of paralogs. This refined view of iron use strategies in diatoms elucidates the history of these adaptations, and provides potential molecular markers for determining the iron nutritional status of different diatom species in environmental samples.

## Introduction

Earth’s early oceans were rich in dissolved ferrous iron, which fostered the evolution of catalytic proteins that relied upon the redox potential of iron [1]. The onset of the Great Oxygenation Event approximately 2.3 Gya [2] caused iron(III) to precipitate out of seawater, transforming iron from a readily-available nutrient to a scarce commodity. Yet the legacy of ferroproteins persists with iron remaining an obligate cofactor of many essential metalloproteins. Photosynthetic organisms have particularly high iron requirements, with about half their total intracellular iron contained within photosynthetic proteins [3–5].

Approximately 20% of global photosynthesis is carried out by marine diatoms [6]. As members of the stramenopiles, diatoms appeared in the fossil record about 190 million years ago [7] and subsequently diverged into four Classes–the more ancient Coscinodiscophyceae and Mediophyceae diatoms (commonly referred to as centric diatoms), and the more recently diverged Fragilariophyceae and Bacillariophyceae diatoms (commonly known as pennate diatoms). Today, diatoms are one of the more species-rich groups of eukaryotic micro-organisms, able to bloom in both iron-rich coastal and iron-poor open ocean environments [8]. Diatoms rely on a diversity of strategies to meet cellular iron demands, including a high-affinity iron uptake system; iron storage capacity; substitutions of iron-requiring proteins with non-ferrous functional equivalents; and mechanisms to mitigate the risk of damage from reactive oxygen species produced in the presence of this redox-active metal [9–11].

The high-affinity reductive uptake system for iron was initially described in yeast [12–14] and consists of a high-affinity ferric reductase (FRE) that dissociates iron(III) from organic ligands; a multi-copper oxidase (MCO) that oxidizes the released iron(II) to iron(III); and an iron permease (FTR) that receives iron(III) from MCO for translocation across the cell membrane. Genes encoding putative ferric reductases [15], the putative multi-copper oxidase [16] and an iron(III) permease [17] have been detected in a limited number of examined diatoms.

Once inside the cell, intracellular concentrations of iron must be tightly regulated to avoid oxidative damage. The best understood system for storing iron is ferritin (FTN), which self-assembles into a multimeric nanocage that sequesters iron(III) within its spherical structure [18]. Initially, genes encoding ferritin were conspicuously absent from the stramenopiles with publicly available whole genome sequences. The discovery in 2009 of *FTN* in a subset of diatoms led to the hypothesis that acquisition of this gene may have facilitated expansion of diatoms into the low-iron environment of the open ocean [19].

Low iron availability in today’s oceans appears to have driven the evolution of proteins that are functionally equivalent to ferroproteins but do not use iron as a cofactor. Common examples are found in the photosynthetic electron transfer chain. The iron-requiring ferrodoxin (encoded by *petF*) can be replaced by flavodoxin (encoded by *FLDA*), which uses flavin, rather than iron, as the redox cofactor [10, 20–22]. Two isoforms encoded by a clade I and a clade II *FLDA* differ in their response to iron availability, with the clade II *FLDA* transcript abundance apparently regulated by iron levels [21]. The gene encoding ferredoxin appears plastid-encoded in most diatoms [23,24], although an evolutionarily recent transfer of the gene to the nucleus was reported for *Thalassiosira oceanica* [25]. A second substitution example is replacement of the iron-requiring cytochrome *c*_*6*_ (CYTC6) with the copper-coordinating plastocyanin (PCYN). Thus far, this replacement has only been observed in *T*. *oceanica* [26], though detection of plastocyanin transcripts have been reported for *Pseudo-nitzschia granii* and *Fragilariopsis cylindrus* [9].

Superoxide dismutases (SODs) combat the formation of reactive oxygen species in the presence of redox-active metals like iron, catalyzing the transformation of O_2_^-^ into molecular oxygen and hydrogen peroxide [27]. Four types of SODs are defined by the use of different metal cofactors: Fe, Mn, Cu-Zn, or Ni. The Fe- and Mn-binding SODs are structurally similar and likely diverged following an ancient gene duplication [27,28]. Cu-Zn- and Ni-utilizing SODs are evolutionarily distinct from each other and from the Fe/MnSODs and may represent convergent evolution of similar function [29]. NiSODs were recently recognized in eukaryotes, having now been identified in the diatom *Phaeodactylum tricornutum* as well as the prasinophytes *Ostreococcus* and *Micromonas* [30–32].

Limited availability of genetic data from diatoms has hindered a better understanding of the influence of various iron utilization strategies on the distribution and ecological success of diatom species. The Marine Microbial Eukaryote Transcriptome Sequencing Project [33] has greatly expanded our knowledge of the breadth and depth of functional genetic diversity of marine microeukaryotes. At the time of this study, the MMETSP consisted of 367 transcriptomes derived from 151 genera and included 77 diatom transcriptomes from 47 species and 31 genera. With this data, in conjunction with existing whole genomes from other microeukaryotes, we examined the diversity and evolutionary history of genes required for different iron metabolic strategies in diatoms.

## Results

### Components of a reductive uptake system appear in the four diatom Classes

**Fig 1:**
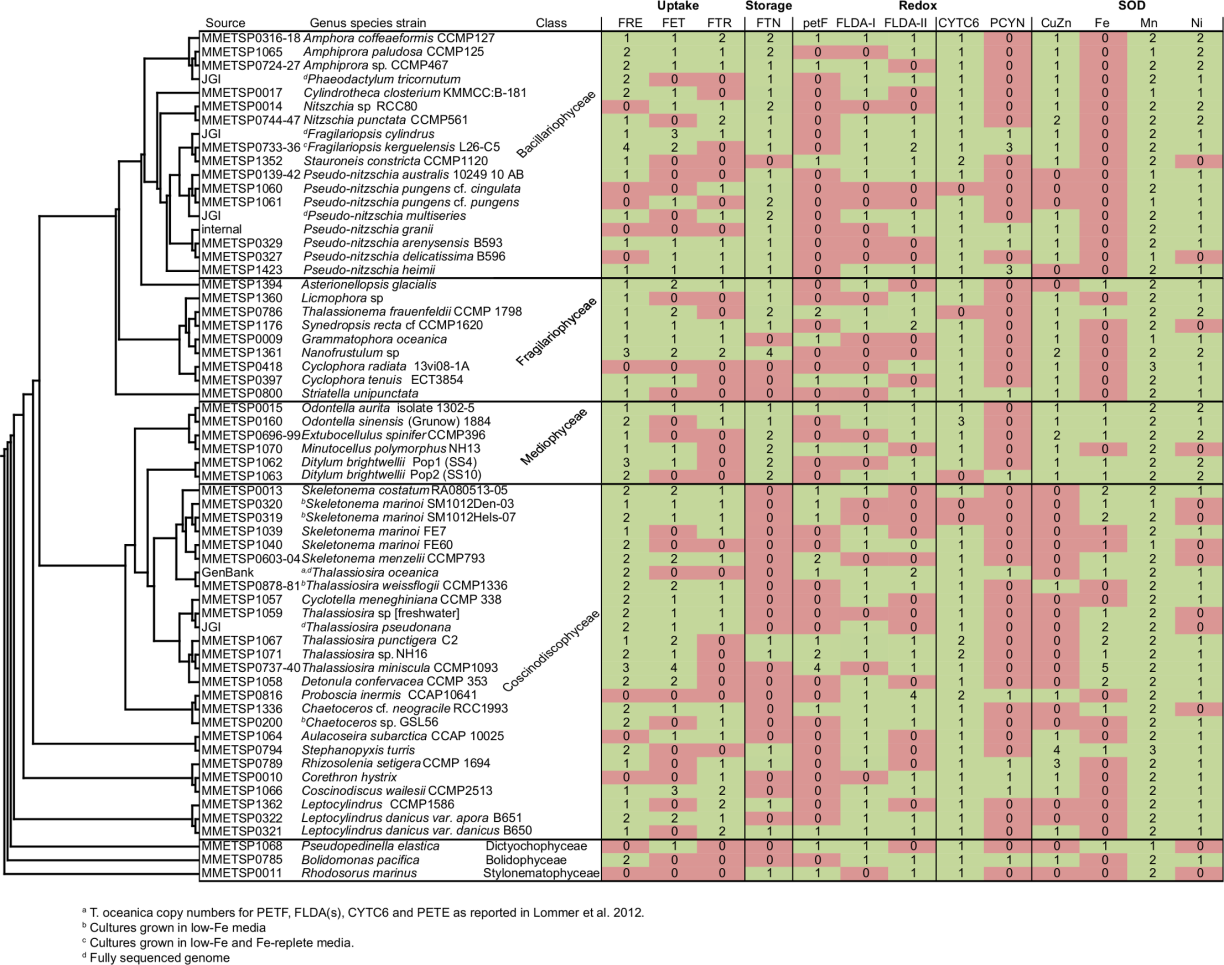
Presence or absence of detected transcripts in diatoms per species or subspecies. Sampled diatoms are shown with three select outgroups. Numbers indicate total unique copy variants as defined by the number of independently clustering paralogs. Abbreviations: ferritin, FTN; flavodoxin, FLAV; ferredoxin, FER; plastocyanin, PCYN; cytochrome c6, CytC6; superoxide dismutase, SOD. Eukaryote-only 18S sequences curated and aligned by SILVA (www.arb-silva.de) were re-aligned with the 18S sequences available for all MMETSP samples and for genomes and non-MMETSP transcriptomes included in our analyses (see Methods). Tree drawn with FastTree. Diatoms all fell within a single clade; all other eukaryote branches except for select outgroups are not shown.

**Fig 2:**
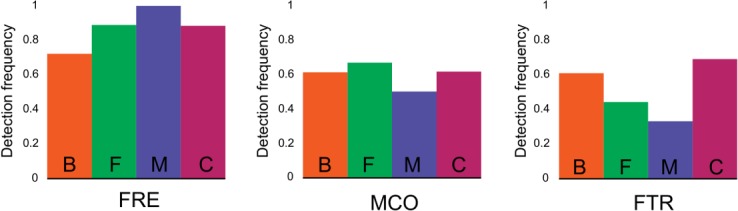
The three components of the reductive uptake system in diatoms. Frequency of detected transcripts for ferric reductase (FRE), multi-copper oxidase (MCO) and iron(III) permease (FTR). Bars show proportion of strains within Bacillariophyceae (B), Fragilariophyceae (F), Mediophyceae (M) and Coscinodiscophyceae (C) where 1 or more transcripts were detected versus total strains.

Transcripts encoding at least one component of the high-affinity iron uptake system—ferric reductase (FRE), multi-copper oxidase (MCO) and/or iron(III) permease (FTR)—were identified in all but 3 diatom species based on the conservation of motifs originally defined in *Saccharomyces cerevisiae* [12–14] (Figs 1 and 2). *Cyclophora radiata*, *Proboscia inermis*, and *Pseudo-nitzschia granii* lacked evidence of transcription of any of the three components, despite the fact that *P*. *granii* was grown under iron-limiting conditions.

Many species transcribed more than one copy of *FRE*, which encodes the ferric reductase (Fig 1). FRE copies were identified based on the presence of key motifs: an FAD binding site [(H-P-F-(S/T)-(V/L/I)] and the NADPH-adenine binding motif [C-(G/A/V)-P]. (S1 Fig). Two deeply branching clades were detected and are referred to here as FRE-I and FRE-II (S2 Fig). The two FRE paralogs identified in *T*. *pseudonana* [15] fall within both clades, while the two paralogs originally identified in *P*. *tricornutum* [15] group within FRE-I; the two FRE paralogs (ScFRE1, 2) identified in *S*. *cerevisiae* [12] group within the FRE-II clade.

Multicopper oxidases (MCOs) are members of a multi-protein family defined by the presence of four conserved copper-binding motifs with Cu-coordinating histidine or cysteine residues. Within this family, ferroxidases (FET) are MCOs with iron(II) oxidizing capacity. The identification of a multicopper oxidase coding gene in *T*. *pseudonana* was based upon homology of copper-binding motifs to yeast FET homologs [15]. The additional diatom sequences identified here contain all four Cu-coordinating motifs, with the exceptions of members of genera *Thalassiosira*, *Skeletonema*, *Odontella*, *Detonula*, and *Thalassionema*, which display incomplete motifs at the second and fourth Cu-coordinating sites observed in MCO paralogs (S3 Fig). Ferroxidase activity of the multicopper oxidase Fet3p in *S*. *cerevisiae* is inferred to be due to three potential residues, E185, D283, and D409 [14]. Alignment of diatom sequences with yeast Fet3p does not reveal conservation of these residues, though other acidic residues are seen adjacent to these sites. Phylogenetic analysis clusters putative MCOs from marine microeukaryotes into multiple clades, likely reflecting functional variants. MCO sequences with known ferroxidase function from four fungi (*Blastobotrys adeninivorans* AFET3, *Cryptococcus neoformans* CNLAC1, *Phanerochaete chrysosporium* MCO-4) join a well-supported clade that includes FET from *T*. *pseudonana* (S4 Fig); an association observed in previous studies [16].

Iron(III) permeases possess dual [REXXE] motifs as defined for *S*. *cerevisiae* (ScFTR) [13]. These residues are maintained in the putative diatom *FTR* transcripts (S5 Fig). Transcripts for a single copy of *FTR* were detected in about half the diatoms across the four Classes, with two paralogs detected in six species (Fig 1). Diatom FTR forms a paraphyletic clade, owing to the inclusion of several non-diatoms, including the ciliate *Tiarina fusus*, the chlorophyte *Ostreococcus mediterraneus*, and the dinoflagellates *Dinophysis acuminata* and *Kryptoperidinium foliaceum*, the latter of which contains a diatom endosymbiont (S6 Fig).

### FTN is present in every diatom Class, with divergent paralogs found

**Fig 3:**
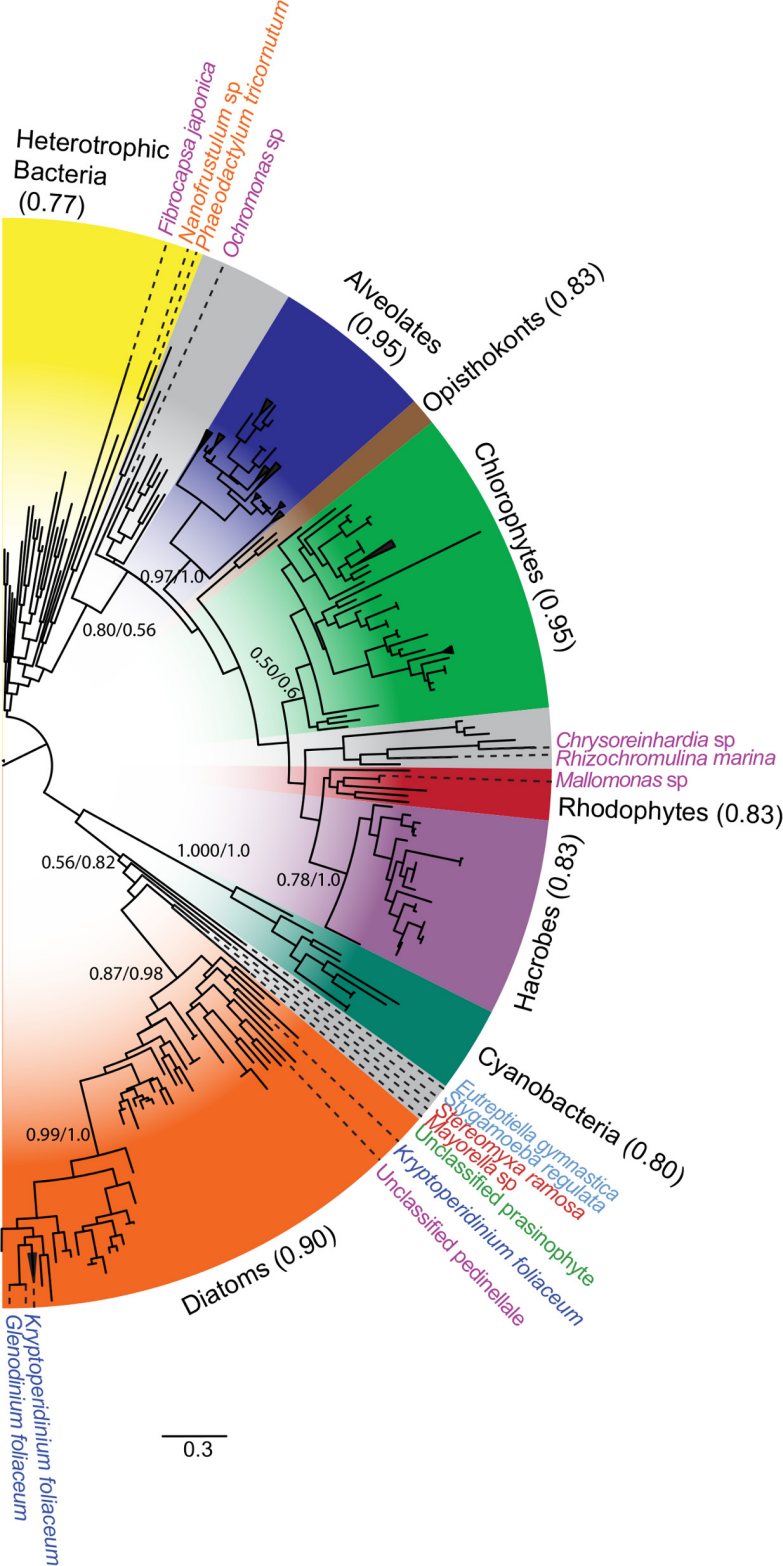
Diatom ferritin is evolutionarily distinct from eukaryotic ferritin. Midpoint-rooted maximum likelihood (ML) tree generated with RAxML using PROTGAMMAWAG model. Numbers at major nodes indicate ratio of bootstrap support values from 1,000 trees; values less than 0.5 removed for clarity. At major nodes, where the branching structure is in agreement with Bayesian inference, posterior probabilities are listed to the right of bootstrap values following a forward slash. Consensus trees were generated with MrBayes v3.1.2 from 1,000,000 generations, with trees sampled every 500 generations. Clades colored by dominant organismal phylogeny: diatoms, orange; mixed clades, grey; cyanobacteria, blue-green; haptophytes and cryptophytes, purple; rhodophytes, red; chlorophytes, green; opisthokonts, brown; alveolates, blue; and heterotrophic bacteria, yellow. Ratio of labeled phylogeny over total taxa in group given beside wedge label. Nodes with multiple nodes from the same taxonomic unit were collapsed. MMETSP and accession IDs for select organisms, clockwise from top: *Fibrocapsa japonica*, MMETSP1339, [CAMERA:0113935616]; *Nanofrustulum* sp., MMETSP1361, [CAMPEP:0202481506]; *Phaeodactylum tricornutum*, [JGI:49895]; *Ochromonas* sp., MMETSP1105, [CAMERA:0173162144]; *Chrysoreinhardia* sp., MMETSP1166, [CAMERA:0118895568]; *Rhizochromulina marina*, MMETSP1173, [CAMERA:0118966858]; *Mallomonas* sp., MMETSP1167, [CAMERA:0182428246]; *Eutreptiella gymnastica*, MMETSP0811, [CAMERA:0174363882]; *Stygamoeba regulata*, MMETSP0447, [CAMERA:0177662056]; *Stereomyxa ramosa*, MMETSP0439, [CAMERA:0174274258]; *Mayorella* sp., MMETSP0417, [CAMERA:0174229308]; unclassified prasinophyte, MMETSP1310, [CAMERA:0119118176]; *Kryptoperidinium foliaceum*, MMETSP0120, [CAMERA:0176017054]; unclassified pedinellale, MMETSP0991, [CAMERA:0171785808]; *Kryptoperidinium foliaceum*, MMETSP0121, [CAMERA:0176108214], [CAMERA:0176054338], [CAMERA:0176293320], and [CAMERA:0176284846]; *Glenodinium foliaceum*, MMETSP0118, [CAMERA:0167862290].

**Fig 4:**
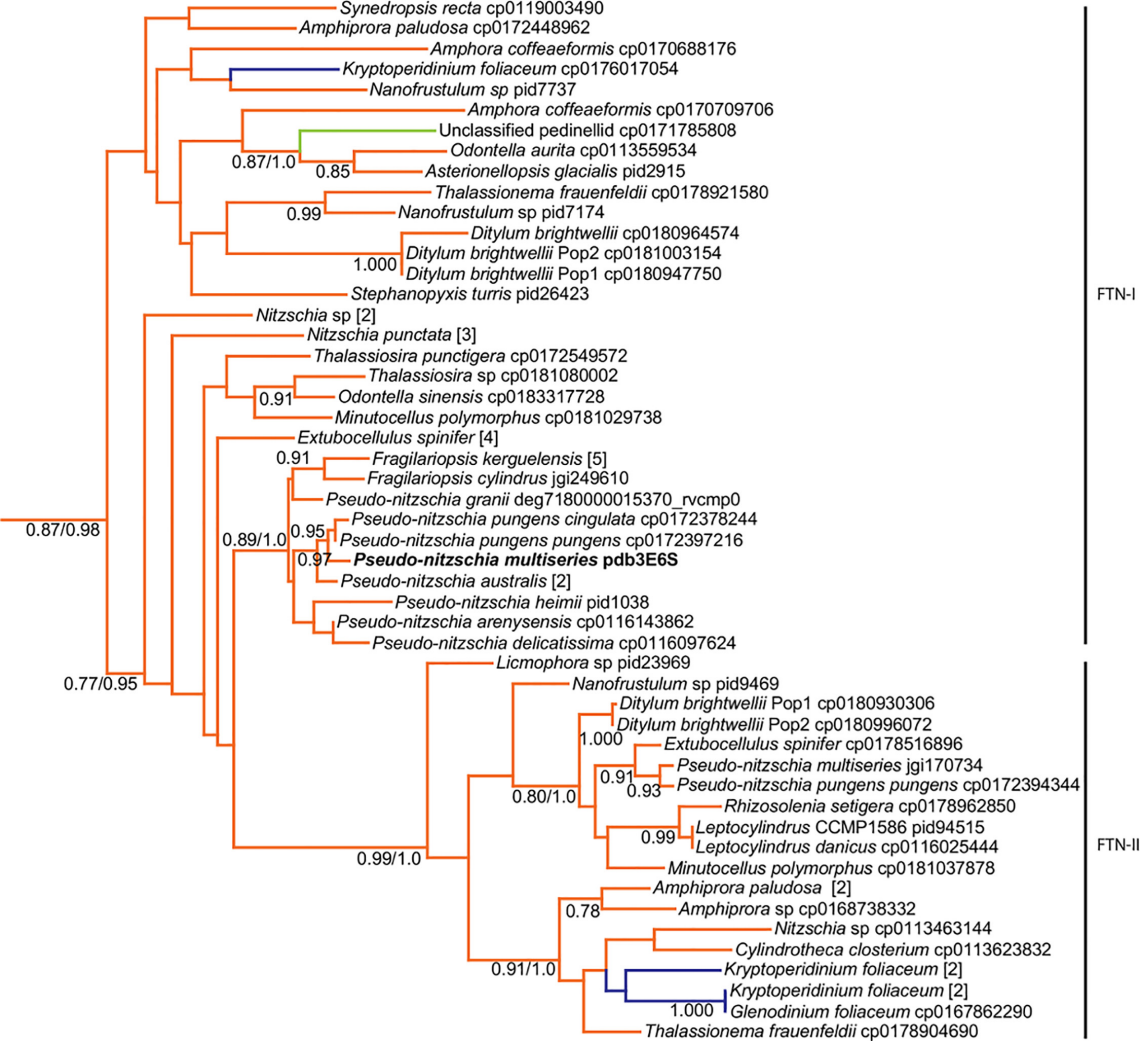
Ferritin phylogenetic tree of diatom clade. Maximum likelihood (ML) tree generated with RAxML using PROTGAMMAWAG model. Numbers beside branches indicate bootstrap support values from 1,000 trees; values under 50 removed for clarity. At major nodes, where the branching structure is in agreement with Bayesian inference, posterior probabilities are listed to the right of bootstrap values following a forward slash. Consensus trees were generated with MrBayes v3.1.2 from 1,000,000 generations, with trees sampled every 500 generations. Branches colored by organismal phylogeny: diatoms, orange; unclassified pedinellid, light green; dinoflagellates, blue. Genus and species are given followed by sequence source and ID. Multiple adjacent tips from the same taxonomic unit were collapsed, with number of members given in brackets.

Ferritin is defined by the presence of key residues at the ferroxidase centers, as confirmed in *P*. *multiseries* FTN by Marchetti et al. [19], and conserved residue pairs shown to be essential for iron release in *Rana catesbeiana* [34]. Putative *FTN* transcripts were detected across the four Classes of diatoms, with at least one copy identified in 33 out of 54 examined species from 21 different genera (Fig 1). The ferroxidase residues are conserved within all diatoms except *P*. *tricornutum* and *Nanofrustulum sp*., which both differ at one residue (S7 Fig). There is less conservation at sites corresponding to iron release in *R*. *catesbeiana* (S7 Fig). Inclusion of bacterial sequences in the ferritin phylogenetic tree resulted in a strongly supported branch composed primarily of diatoms and cyanobacteria, separate from other eukaryotes and heterotrophic bacteria (Fig 3). The diatom clade contains sequences from all four diatom Classes (Figs 1 and 4). A subset of diatom ferritin, henceforth referred to as Group II ferritin (FTN-II), is supported by high bootstrap support (Fig 4), and shows distinct residue differences at the C-terminus from Group I ferritin (FTN-I) (S7 Fig). Transcripts from two dinoflagellates and from an unclassified pedinellid silicoflagellate grouped within the diatom clade (Fig 4). The dinoflagellates *Glenodinium foliaceum* and *Kryptoperidinium foliaceum* both transcribed sequences that grouped closely with sequences from *Nitzschia* and *Cylindrotheca* (Bacillariophyceae) (Fig 4). The putative ferritins from two diatoms–one identified in the *P*. *tricornutum* genome and one of the four putative ferritins from *Nanonofrustum–*were most similar to each other and grouped with heterotrophic bacteria rather than within the diatom clade (Fig 3).

Putative ferritins derived from 5 non-diatom stramenopile genera were distributed across the ferritin tree in a manner that did not match their 18S rDNA phylogeny (Fig 3), including the presence of the silicoflagellate sequence within the diatom cluster (Fig 4). Ferritin from the pelagophyte *Chrysoreinhardia* sp and the dictyophyte *Rhizochromulina marina* clustered within a clade otherwise composed primarily of haptophytes and chlorophytes (Fig 3). Similarly, a sequence from the synurophyte *Mallomonas* sp clustered with a clade of red algae and a sequence from the chrysophyte *Ochromonas* sp.

### Redox protein pairs Ferredoxin/Flavodoxin

**Fig 5:**
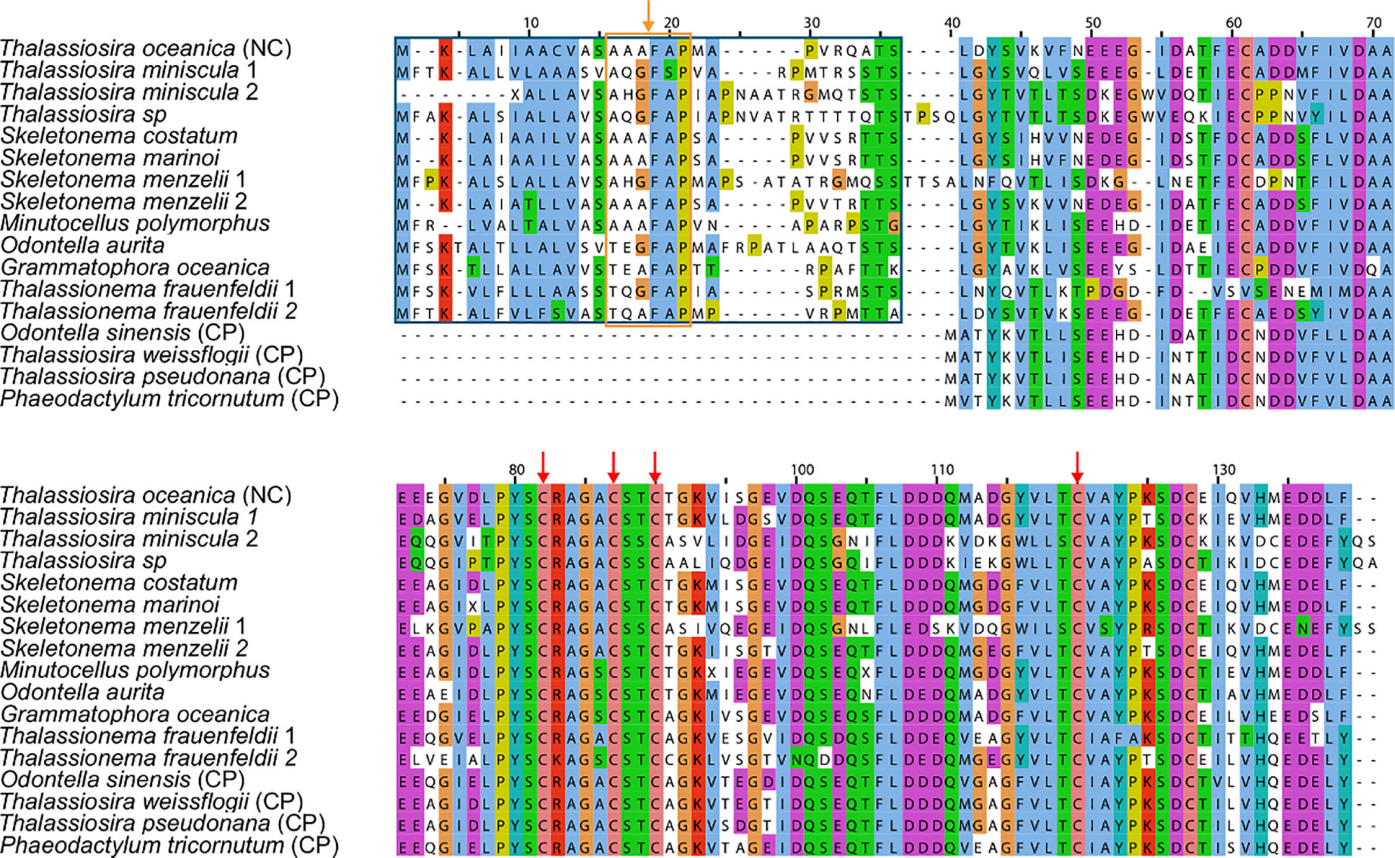
Several species of diatoms show a putative plastid localization sequence on *petF*. Representative *PETF* sequences that possess putative chloroplast transit peptides as identified in the nuclear-encoded *T*. *oceanica PETF* (top, NC) and plastid-encoded *petF* from *O*. *sinensis*, *T*. *weissogii*, *T*. *pseudonana* and *P*. *tricornutum* (bottom, CP). Residues corresponding to *T*. *oceanica* transit peptides highlighted in blue box. The orange box marks the ASAFAP motif, with potential cleavage site between A/G and F marked by an orange arrow. Red arrows mark Fe-coordinating cysteine residues. Twenty-eight unmatched amino acids unique to the N-terminus of *Grammatophora oceanica* (MMETSP0009) were omitted for brevity. MMETSP and Accession IDs: *Thalassiosira oceanica*, [GenBank:EJK54785.1]; *Thalassiosira miniscula* 1, MMETSP0737, [CAMERA:0183720344]; *Thalassiosira miniscula* 2, MMETSP0737, [CAMERA:0183726686]; *Thalassiosira* sp., MMETSP1071, [CAMERA:0181112606]; *Skeletonema costatum*, MMETSP0013, [CAMERA:0113387486]; *Skeletonema marinoi*, MMETSP0319, [CAMERA:0115919778]; *Skeletonema menzelii* 1, MMETSP0604, [CAMERA:0183674844]; *Skeletonema menzelii*, MMETSP0603, [CAMERA:0183648216]; *Minutocellus polymorphus*, MMETSP1070, [CAMERA:0181038080]; *Odontella aurita*, MMETSP0015, [CAMERA:0113537566]; *Grammatophora oceanica*, MMETSP0009, [CAMPEP:0194032050]; *Thalassionema frauenfeldii*, MMETSP0786, [CAMERA:0178915392]; *Thalassionema frauenfeldii*, MMETSP0786, [CAMERA:0178916612]; *Odontella sinensis* (CP), [Swiss-Prot:P49522]; *Thalassiosira weissflogii* (CP), [Swiss-Prot:O98450]; *Thalassiosira pseudonana* (CP), [GenBank:YP_874492], *Phaeodactylum tricornutum* (CP), [GenBank:YP_874403].

The gene encoding ferrodoxin (*petF*) has been previously observed to be plastid-encoded in diatoms, although a nuclear-encoded version of *petF* was recently detected in *T*. *oceanica* [25]. In our study, transcripts encoding ferredoxin were identified in about a third (17) of the examined species of diatoms (Fig 1). The ferrodoxin sequences all contain the four cysteine residues implicated in 2Fe-2S binding in ferredoxin from the green alga *Chlorella fusca* [35] at Cys37, Cys42, Cys45 and Cys75 (Fig 5). The *petF* transcripts from nine species display a putative plastid localization signal with accepted variations in the “ASAFAP” motif [25,36] suggesting a migration of the plastid genome-encoded *petF* to the nuclear genome (Fig 5). The majority of putative *petF* transcripts that encode a plastid localization signal cluster on a phylogenetic tree with the known diatom plastid-encoded *petF* (S8 Fig).

Diatoms possess two flavodoxin isoforms—‘long’ flavodoxin (*FLDAl)* and ‘short’ flavodoxin (*FLDAs)*—that cluster into phylogenetic clades I and II, respectively [21] (S9 Fig). Transcription of clade II *FLDAs* from *Thalassiosira weissflogii* and *Thalassiosira oceanica* is responsive to iron levels and results in production of a flavodoxin that putatively replaces ferredoxin under iron stress [10,21]. The function of clade I flavodoxin is currently unknown. Transcripts for the clade I *FLDAl* were detected in all four diatom Classes, but with more frequent detection in the Coscinodiscophyceae and Mediophyceae (Fig 1). Transcripts for the clade II *FLDAs* were detected in over half the examined species from all four major diatom Classes regardless of experimental conditions, most of which included iron-replete media. Three species—*Proboscia inermis*, *Synedropsis recta*, and *Fragilariopsis kerguelensis*—transcribed 2 or more distinct paralogs of clade II *FLDAs* (S9 Fig).

### Redox protein pairs Cytochrome c_6_/ Plastocyanin

**Fig 6:**
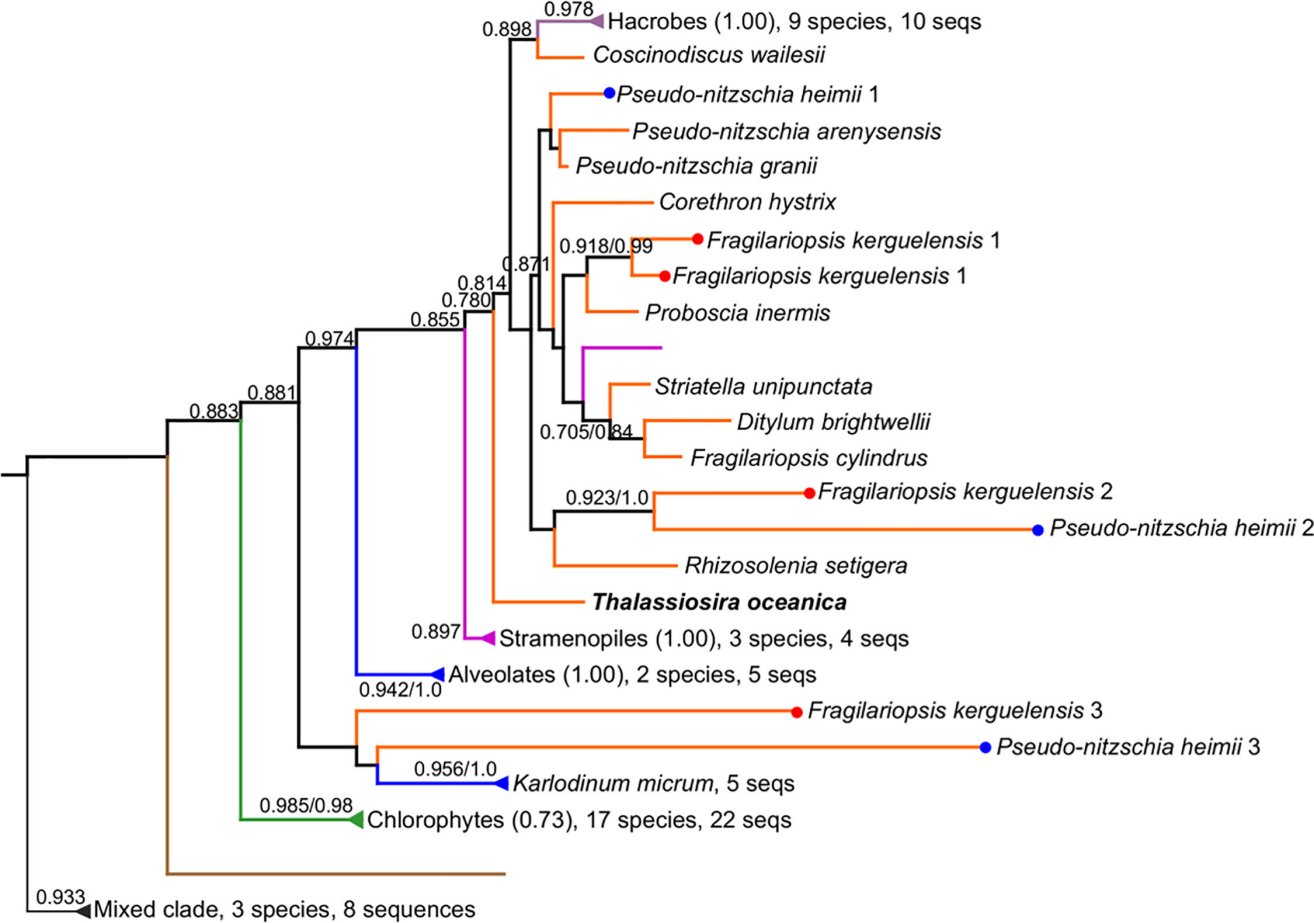
Relationship of diatom PCYN to other phytoplankton. Midpoint-rooted approximately-maximum-likelihood tree of putative and known PCYN. Support values shown for deep nodes, with values under 0.5 removed for clarity. Branches outside of diatom clade are collapsed; with dominant phylogenetic composition, ratio of dominant taxa to total taxa, number of species in the collapsed clade, and number of sequences. At major nodes, where the branching structure is in agreement with Bayesian inference, posterior probabilities are listed to the right of bootstrap values following a forward slash. Consensus trees were generated with MrBayes v3.1.2 from 2,400,000 generations, with trees sampled every 500 generations. One representative is shown from groups sharing greater than 95% similarity in aligned sequence identity. Branches colored by organismal phylogeny: diatoms, orange; chlorophytes, green; haptophytes and cryptophytes, lavender; non-diatom stramenopiles, magenta; alveolates, blue; opisthokonts and amoebozoa, brown, mixed clades, gray. Previously identified PCYN from *Thalassiosira oceanica* noted in bold. Distinct *P*. *heimii* and *F*. *kerguelensis* paralogs shown with red or blue dots, respectively. Top to bottom, diatom labels: *Coscinodiscus wailesii*, [CAMERA:0172483904]; *Pseudo-nitzschia heimii*, [CAMPEP:0197183406]; *Pseudo-nitzschia arenysensis*, [CAMERA:0116141514]; *Pseudo-nitzschia granii*, IH deg7180000014200 frame0; *Corethron hystrix*, [CAMERA:0113306274]; *Fragilariopsis kerguelensis*, [CAMERA:0170793268]; *Fragilariopsis kerguelensis*, [CAMERA:0170902168]; *Proboscia inermis*, [CAMERA:0171306160]; *Striatella unipunctata*, [CAMERA:0118690216]; *Ditylum brightwellii*, [CAMERA:0180970060]; *Fragilariopsis cylindrus*, [JGI:272258]; *Fragilariopsis kerguelensis*, [CAMERA:0170771410]; *Pseudo-nitzschia heimii*, [CAMPEP:0197180752]; *Rhizosolenia setigera*, [CAMERA:0178972290]; *Thalassiosira oceanica*, [EMBL:D2Z0I2]; *Fragilariopsis kerguelensis*, [CAMERA:0170889260]; *Pseudo-nitzschia heimii*, [CAMPEP:0197182106].

Transcripts encoding cytochrome c_6_ (*CYTC6)* were detected in at least one representative transcriptome from each of the examined diatom species, with the exception of *Thalassionema frauenfeldii* (Fig 1). The copper-containing redox protein plastocyanin can substitute for cytochrome c_6_ in the green alga *Chlamydomonas* [37]. Transcripts encoding putative plastocyanin (*PCYN*) were detected in at least one species from each of the major diatom Classes (Fig 1). *Pseudo-nitzschia heimii* and *Fragilariopsis kerguelensis*, both open-ocean species, possess three distinct copies of putative *PCYN* in contrast to other species in which only one variant was detected. All identified sequences maintain conservation of the canonical Cu-coordinating residues demonstrated in *Populus nigra* [38], with the notable exception of one copy from *P*. *heimii*, which does not conserve 2 of the 4 positions (S10 Fig). The diatom sequences cluster as a monophyletic clade that appears to share a common ancestor with other chromealveolates. Both *F*. *kerguelensis* and *P*. *heimii* exhibit one copy that does not cluster with other diatom sequences (Fig 6).

### Preferential transcription of FeSOD in basal diatoms; Cu-ZnSOD in derived diatoms

**Fig 7:**
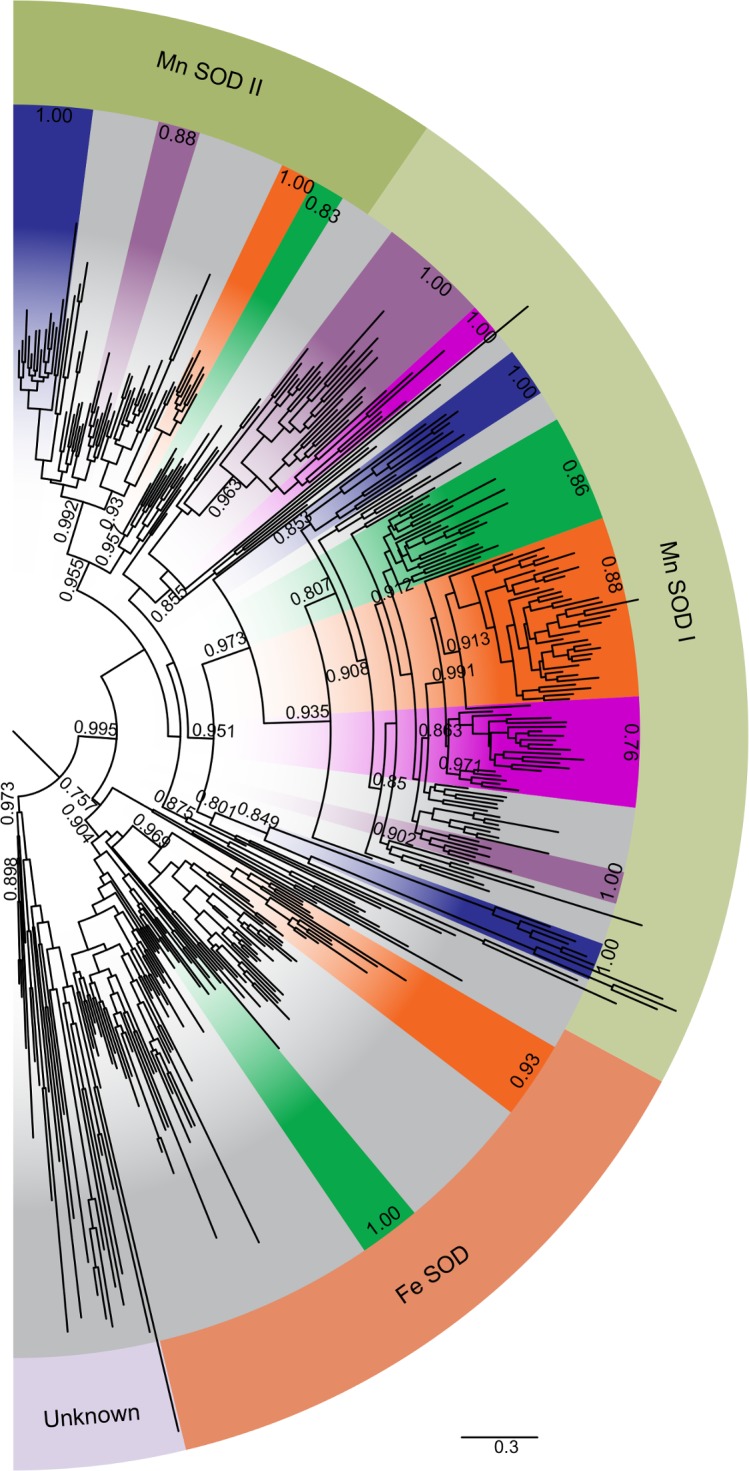
The evolutionary relationship of Fe and Mn superoxide dismutase genes. Midpoint-rooted approximately-maximum-likelihood tree of putative and known Fe and Mn SOD amino acid sequences. Outer border wedges indicate putative function as FeSOD, MnSOD I, or MnSOD II. Inner border wedges indicate dominant phylogenetic composition: diatoms, orange; mixed clades, grey; haptophytes and cryptophytes, purple; chlorophytes, green; alveolates, blue; and non-diatom stramenopiles, magenta. Decimals at edges of inside wedges give ratio of labeled phylogeny over total taxa in group. Bootstraps omitted for clarity. One representative is shown from groups sharing greater than 95% similarity in aligned sequence identity.

**Fig 8:**
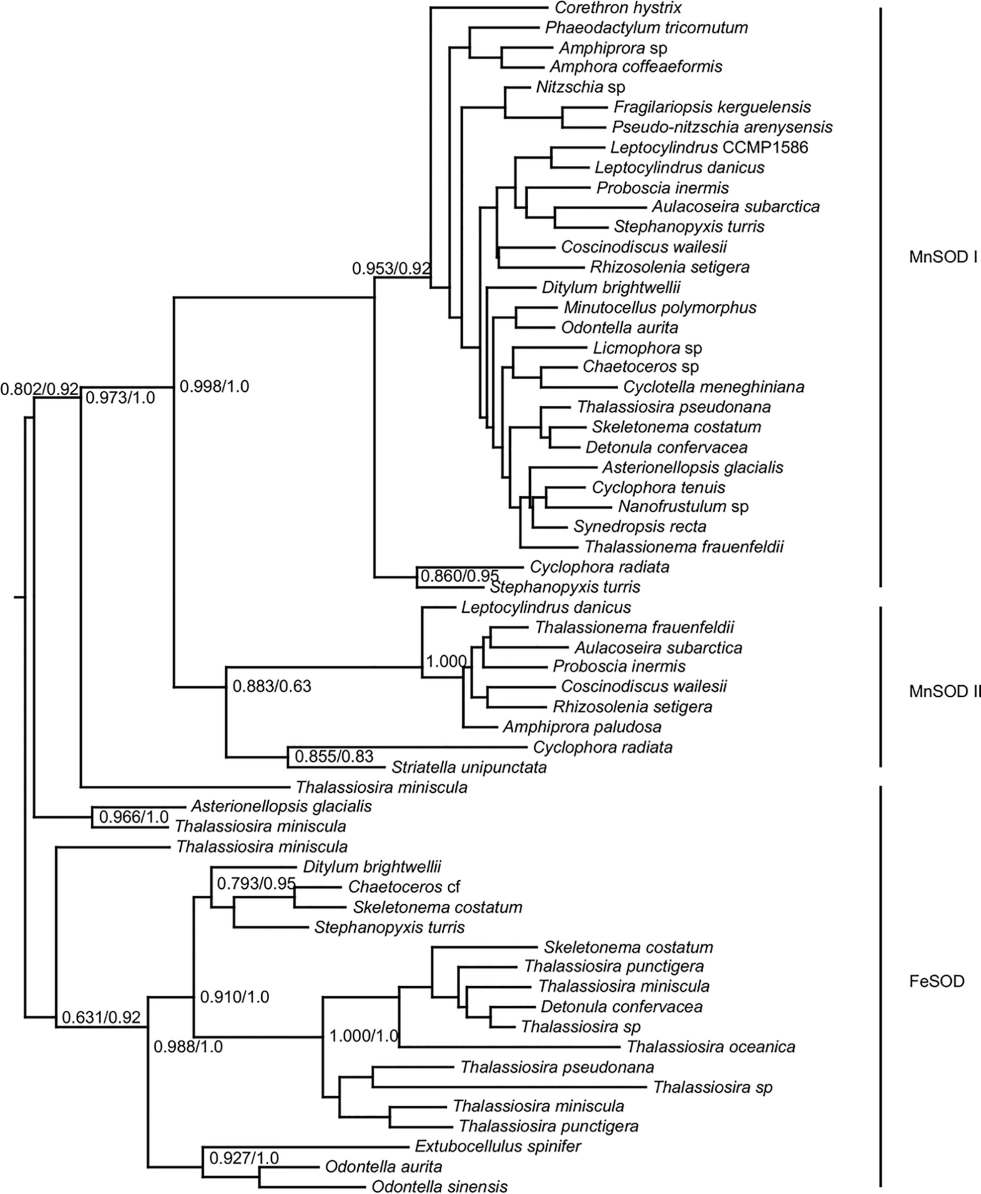
Phylogenetic tree of Fe and Mn SOD from diatoms only. Midpoint-rooted approximately-maximum-likelihood tree of putative and known Fe and Mn SOD amino acid sequences. Sequences with less than 95% similarity in aligned residues shown. Node support values are calculated from 1,000 resamples, only values over 0.5 are shown. At major nodes, where the branching structure is in agreement with Bayesian inference, posterior probabilities are listed to the right of bootstrap values following a forward slash. Consensus trees were generated with MrBayes v3.1.2 from 1,700,000 generations, with trees sampled every 500 generations.

Transcripts encoding iron and manganese superoxide dismutase (FeSOD and MnSOD) were distinguished by the presence of key metal-coordinating residues (FeSOD: Q-77 and A-146, MnSOD: G-77 and Q-146) (S11 Fig), [39] and were identified in a wide variety of eukaryotic groups (Fig 7). *MnSOD* transcripts were detected for all examined diatoms, with most diatoms transcribing two paralogs (Fig 1). Transcripts for the closely related *FeSOD* were more taxonomically restricted. About half the Coscinodiscophyceae transcribed one or more *FeSOD* paralogs and all but one species of Mediophyceae transcribed a single copy. Less than a quarter of the more recently diverged Fragilariophyceae and none of the Bacillariophyceae transcribed *FeSOD* homologs. The two *MnSOD* paralogs are separated from each other and from *FeSOD* by well-supported branches (Fig 8).

Cu-ZnSOD functionality in spinach (*Spinacia oleracea*) has been attributed to Cu ligands His46, His48, His63, His120 and Zn ligands His63, His71, His80, and Asp83 [40]; these residues are also conserved in yeast Cu-ZnSOD [41]. Full conservation of residues corresponding to His46, His48, His63, His80 and His120 in spinach was identified within diatoms only in *Rhizosolenia setigera* (MMETSP0789), *Stephanopyxis turris* (MMETSP0794), *Nitzschia punctata* (MMETSP0744-47), and *Nanofrustulum* sp (MMETSP1361), representing three separate Classes. Within a majority of the diatom sequences, only the Zn ligand sites His71 and Asp83 were fully conserved (S12 Fig). Although most retrieved diatom sequences do not maintain all the canonical His residues, they display overall sequence homology to either the spinach (42–52% identity) or yeast (39–70% identity) Cu-ZnSOD.

The distribution of putative Cu-Zn versus FeSODs in the examined diatoms displayed opposing trends. The basal Coscinodiscophyceae and Mediophyceae lineages more frequently displayed transcripts for the FeSODs, although transcripts corresponding to Cu-ZnSODs were also detected throughout these two Classes (Fig 1). In contrast, the more recently diverged Fragilariophyceae showed a bias towards Cu-ZnSOD rather than FeSOD transcripts; only *Asterionellopsis glacialis* did not reveal a Cu-ZnSOD, instead transcribing an FeSOD, while *Thalassionema frauenfeldii* displayed both forms. No FeSOD transcripts were detected within the 18 examined species of Bacillariophyceae; all instead transcribed the Cu-ZnSOD.

### NiSOD transcription is common in diatoms

**Fig 9:**
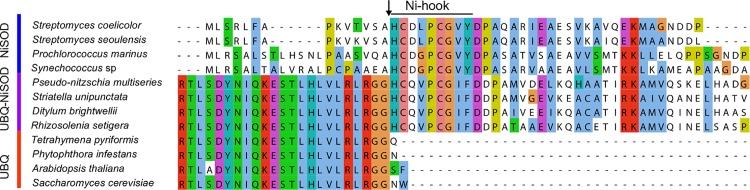
The Ubiquitin-NiSOD fusion protein. Section of a multiple sequence alignment of verified NiSOD, verified ubiquitin (UBQ), and putative UBQ-NiSOD fusion genes. Trimmed to show terminal 23 residues of UBQ, and first 29 residues of NiSOD. Accession numbers: Bacterial NiSOD (SodN) sequences (top four rows, blue side bar: *Streptomyces coelicolor*, [Swiss-Prot:P80735]; *Streptomyces seoulensis*, [Swiss-Prot:P80734]; *Prochlorococcus marinus*, [Swiss-Prot:Q7V8K4]; *Synechococcus* sp. WH8102, [Swiss-Prot:Q7U5S1]) aligned with putative UBQ-NiSOD fusion protein from one representative of each diatom class (middle four rows, purple bar: *Pseudo-nitzschia multiseries*, [JGI:206387]; *Striatella unipunctata*, MMETSP0800, [CAMERA:0118699990]; *Ditylum brightwellii*, MMETSP1063, [CAMERA:0180982606]; *Rhizosolenia setigera*, MMETSP0789, [CAMERA:0178952988]) and the terminal UBQ from four model eukaryotes (bottom four rows, red bar: *Tetrahymena pyriformis*, [Swiss-Prot:P0CG82]; *Phytophthora infestans*, [Swiss-Prot:P22589]; *Arabidopsis thaliana*, [Swiss-Prot:Q1EC66]; *Saccharomyces cerevisiae*, [Swiss-Prot:P0CG63]). Black arrow indicates cleavage site for NiSOD in bacteria, cleavage site for UBQ in eukaryotes, and proposed cleavage site for UBQ-NiSOD fusion protein.

A putative NiSOD-encoding transcript (*NiSOD*) was detected in forty-five (85%) of the queried diatom species (Fig 1), with the majority of detected NiSODs encoded immediately downstream of ubiquitin by an ancient UBQ-NiSOD fusion gene (Fig 9, S13 Fig). These putative NiSODs conserve the Ni-hook motif required for superoxide dismutase activity [42]. Nine species transcribed a second NiSOD paralog without an apparent fusion to UBQ. A majority of UBQ-lacking NiSOD transcripts from across the marine microeukaryotes branch more deeply than those NiSODs that possess the UBQ presequence suggesting that the presequence is a derived state (S13 Fig).

## Discussion

We used the sequence data generated for the Marine Microbial Eukaryote Transcriptome Sequencing Project (MMETSP) [33] to investigate the prevalence of select iron metabolism genes across diatoms. Previous research on the metabolic capabilities of these organisms had necessarily been limited by sequence data derived from a relatively low number of samples. Here, we re-evaluate previous hypotheses in light of the expanded resolution and breadth of organismal diversity enabled by the MMETSP. Of the 77 examined diatom transcriptomes, nine were derived from four diatom species grown under potentially iron-limiting conditions (media with less than 60 nmol Fe L^-1^): *Thalassiosira weissflogii* (MMETSP0878-0881), *Chaetoceros* sp. (MMETSP0200), *Fragilariopsis kerguelensis* (MMETSP0735, 0736), and *Skeletonema marinoi* (MMETSP0319, 0320). A publicly available transcriptome of *Pseudo-nitzschia granii* grown under iron-limiting conditions was also included. Despite the majority of cultures having been grown in iron-replete conditions, we detected transcripts for genes more commonly associated with iron limitation. Given this bias in growth conditions, it is important to note that the absence of detected transcripts in any given sample may mean that the culture conditions tested did not result in significant transcription of a gene. Due to differences in culturing conditions inherent to a distributed collaborative effort, we cannot confidently compare quantitative read counts of genes between samples. Therefore, these data provide information about the presence, but not absence or differential transcription, of particular sequences. Taken together, the data reveal a more complete picture of the distribution of iron metabolism genes across taxa, challenging assumptions and providing insight into gene origins, copy number, and divergent functions and affinities.

### Revisiting hypotheses of lateral gene transfer

Lateral gene transfer (LGT) in eukaryotes is a recognized mechanism for the gain of new biochemical functions, increasing the potential for expansion into new ecological niches. Accurate detection of LGT, however, requires significant representation of sequences across a comprehensive taxonomic range [43]. The MMETSP data reveal one case that supports, with modifications, a hypothesis of LGT origin (*FTN*), a second case that expands upon a hypothesis of endosymbiotic gene transfer (*petF*), and two cases where previously hypothesized LGT instead likely reflects vertical inheritance within diatoms (*NiSOD* and *PCYN*).

Prior to the MMETSP, the handful of available ferritin sequences from diatoms came from pennate lineages. *FTN* sequences from pennate diatoms were separated from the other eukaryotes by long branches on a phylogenetic tree. The dissimilarity of the pennate diatom *FTN* from other eukaryotes, together with its apparent absence from other stramenopiles supported the original hypothesis of LGT acquisition of *FTN* in pennates [19]. The MMETSP data serve to extend and modify the story by showing *FTN* transcription in all four extant diatom Classes (Fig 1), while remaining largely absent from other stramenopiles. The additional diatom sequences identified here confirm that diatom ferritin does not branch with other eukaryotic ferritins and instead branches with cyanobacterial true ferritin, which is believed to also possess iron storage functionality, suggesting an ancient acquisition of *FTN* from this group (Fig 3). Two putative diatom *FTN* sequences appear more closely related to those from heterotrophic bacteria rather than photosynthetic cyanobacteria. One of the “bacteria-like” sequences is found in the *Phaeodactylum tricornutum* whole genome sequence [44] and groups closely with a sequence from a different diatom species, *Nanofrustulum sp*., suggesting that these sequences may have been acquired as separate LGT events from closely related heterotrophic bacteria (Fig 3).

Embedded within the clade of diatom ferritins are sequences derived from two dinoflagellates and one silicoflagellate (Fig 4). The two dinoflagellates (*G*. *foliaceum* and *K*. *foliaceum*) are closely related ‘dinotoms’ whose plastids are derived from a diatom endosymbiont [45], likely a member of the Bacillariophyceae [46]. The close affiliation of the dinotom *FTN* sequences with *Nitzschia*, *Cylindrotheca* and *Nanofrustulum* suggest that they are derived from their diatom endosymbiont rather than the dinoflagellate host. In contrast, the unrelated silicoflagellate may have acquired ferritin through the phagotrophy of a diatom, specifically a member of the Mediophyceae.

The absence of *FTN* in some diatoms, such as certain members of *Thalassiosira*, may be tolerated due to the presence of an alternative, non-ferritin based iron storage system. Such a system has been proposed for both the brown alga *Ectocarpus siliculosis* [47] and the *Thalassiosiroid* diatoms [48], both of which lack a known genomic copy of *FTN* and appear to store iron in mineralized clusters.

Similar to LGT, endosymbiotic gene transfer (EGT) is the acquisition of genetic material from outside the host genome, in this case from an endosymbiont or endosymbiotically-derived organelle. We identified evidence for transfer of *petF*, which encodes ferredoxin, from the plastid to the nuclear genome. *petF* transcripts from multiple species across classes were detected despite the use of mRNA isolation protocols that bias against plastid encoded transcripts. A majority of these transcripts appear to encode a plastid-targeting peptide (Fig 5) similar to that detected for the *T*. *oceanica* variant [25], including conserved motifs recognized for plastid localization [36]. Transfer of *petF* from the plastid to the nucleus may have occurred several times, or alternately, this transfer may have occurred once in an ancestral diatom with subsequent loss in some descendant lineages (S8 Fig). Nuclear regulatory control of *PETF* has been suggested to provide a more nuanced response to iron availability in *T*. *oceanica* [25], and this may reflect a more ancestral method of acclimation to trace metal availability in diatoms.

In contrast to the incorporation of *FTN* and *petF* into the diatom nuclear genome by LGT and EGT, respectively, two genes with hypothesized recent LGT origins appear to instead have roots deeper in the chromalveolate lineage. *NiSOD* and *PCYN* homologs are broadly distributed throughout diatoms, but unlike *FTN* they follow organismal phylogeny, clustering with other chromalveolates (S13 Fig, Fig 6). The gene encoding nickel superoxide dismutase (*NiSOD*) was originally hypothesized to have arisen relatively recently in eukaryotes via a lateral gene transfer from bacteria to prasinophytes (*Ostreococcus*) based on available molecular data at the time [31,32]. More recently, a *NiSOD* homolog was found in the diatom *Phaeodactylum tricornutum* [30]. Similarly, the apparent scarcity of *PCYN*, only previously identified in *T*. *oceanica* [26] and *Fragilariopsis cylindrus* [49] was hypothesized to reflect LGT events in select diatom species [50]. Our analyses demonstrate that homologs of both genes are present in every class of diatoms and many of the major branches of the chromalveolates, which supports vertical, rather than lateral, inheritance of these genes.

### The multi-copy nature of iron genes contributes to interspecies variability

Iron metabolism proteins in diatoms appear to be encoded primarily by multi-gene families, presumably resulting in proteins with divergent functions. For example, in many diatom species, multi-copy gene families were detected for two of the iron uptake system proteins–multicopper oxidase and ferric reductase. Multi-copper oxidases include members with ferroxidase and cuprous oxidase activity, and share sequence similarities with laccases [14]. The residues implicated in ferroxidase activity in yeast [14] are not maintained in diatoms, although ferroxidase activity has been demonstrated *in vivo* with *T*. *oceanica* [16]. Similarly, the vast majority of diatoms harbor multiple copies of putative diatom ferric reductase genes (*FRE*). This variability in copy number could allow for neofunctionalization of paralogs, perhaps resulting in separate metabolic functions.

An ancient duplication event appears to have led to at least two distinct paralogs of diatom ferritin, with a well-supported subset, *FTN-II*, showing distinct divergence of residues on the C-terminus of the predicted translation (Fig 4, S7 Fig). Mammals possess a light chain and heavy chain of ferritin: the heavy chain oxidizes iron(II), while the light chain fosters ferrihydrite nucleation, having lost the capacity to oxidize iron [51]. Whether or not the two paralogs of diatom ferritin form a complex or demonstrate functional differences is unknown, as only one paralog from *P*. *multiseries* (PID 237986) from *FTN-I* has been characterized [19,52].

Three putative plastocyanin paralogs were detected in *Fragilariopsis kerguelensis* and *Pseudo-nitzschia heimii*, both of which are found in low-iron open ocean regions. These alternate paralogs possess the canonical Cu-coordinating residues for *Populus nigra* plastocyanin [38], with one notable exception in *P*. *heimii* (*PCYN1*), which has His37Gln and Cys84Ser substitutions at the Cu-coordinating site relative to *P*. *nigra* PCYN, conceivably leading to altered binding capacity or neofunctionalization (S10 Fig). Characterization studies would be useful to determine the role of these additional copies of *PCYN* in *P*. *heimii* and *F*. *kerguelensis*, and to assess whether they confer an advantage in low-iron regimes.

### Multiple superoxide dismutase metalloforms illustrate adaptive preferences for different transition metals

Diversification of function through gene duplications is exemplified with the SODs. The biochemical importance of SOD is underscored by the presence of multiple isozymes. At least two different metalloforms of SODs were detected in every species of diatom (Fig 1). The gene encoding manganese superoxide dismutase (*MnSOD*), a metalloform that has been suggested to substitute for FeSODs under iron-limiting conditions [26], appears to have duplicated early in eukaryotic history, with most diatoms transcribing two or more paralogs (Fig 1, Fig 8). The common detection of *NiSOD* transcripts implies an important, constitutive role for the NiSOD as well. Most *NiSOD* transcripts identified here are fused to an ubiquitin-coding sequence (S13 Fig). In yeast, post-translational cleavage of ubiquitin-fusion proteins is performed by at least four ubiquitin-specific proteases [53]. Similar cleavage by ubiquitin-specific proteases of the NiSOD fusion protein would provide a mechanism for the immediate activation of the Ni-hook motif and suggest the possibility that UBQ regulatory pathways may control activated NiSOD protein abundance. Unlike NiSOD from bacteria, the complete putative NiSOD homolog from these eukaryotes has yet to be fully functionally characterized, although SOD functionality has been demonstrated in oligopeptide maquettes [31,54].

In contrast to the near ubiquity of *Mn* and *NiSOD* transcripts, *Fe* and *Cu-Zn SODs* displayed distinctive patterns. *FeSOD* was not detected in any members of the Bacillariophyceae, the most derived class of diatoms. Instead, *FeSOD* transcripts were more commonly detected as two distinct copies in members of the most ancient class of diatoms, the Coscinodiscophyceae (Fig 1). The *Cu-ZnSOD*s displayed an opposite trend to the *FeSOD*s, with transcripts more frequently detected in the more derived diatoms (Fig 1). The apparent preference of pennate diatoms for *Cu-ZnSOD* in Fe-replete media, and their parallel lack of *FeSOD* transcripts, suggests a permanent shift in metal-use priorities for this group of diatoms. While previous studies have emphasized the role of Fe and Cu-Zn SODs in diatoms, our analyses suggest that they may play an accessory role to the dominant Mn and Ni metalloforms.

## Conclusions

The data presented in this study provides a revised perspective on the distribution and prevalence of key genes involved in iron metabolism in marine diatoms. The presence of transcripts encoding the three elements of the reductive uptake system (*FRE*, *MCO*, *FTR*) throughout the diatom lineage is evidence that this system has been evolutionarily conserved. We report that ferritin (*FTN*) coding genes are present in ancient diatoms, comprising a lineage distinct from canonical eukaryotic *FTN*. Additionally, two *FTN* paralogs are present in many diatoms, with one divergent clade displaying distinct differences on *in silico* translated C-terminal residues. The distribution of transcripts encoding the non-ferrous electron carriers plastocyanin (*PCYN*) and flavodoxin (*FLDA*) suggests the potential use of alternative redox metal strategies in a greater range of species than previously observed. Homologs of all four superoxide dismutase (SOD) metalloforms were found, illustrating the potential for adaptive use of different isozymes to ensure protection against oxidative damage in the face of metal scarcity.

Based on our analyses, much of the physiological diversity found in diatoms appears to come from gene duplications and subsequent divergence. In the majority of cases, we found species harbor multiple paralogs suggestive of functional diversification and lending insight into the adaptable nature of diatoms that may have contributed to their expansion into so many habitats.

## Methods

### Datasets

Processing and sequencing of samples was performed at the National Center for Genome Resources (NCGR) as part of the Marine Microbial Eukaryote Transcriptome Sequencing Project (MMETSP) [33] and all sequence and metadata were retrieved through the CAMERA portal [55]. The assembly pipeline for the MMETSP transcriptomes is described in more detail at the project website.

The following datasets were used for comparative analyses: 360 MMETSP transcriptomes publicly available as of November 4^th^, 2013; seven transcriptomes from diatoms cultured in-house for the MMETSP which were not public on CAMERA as of November 4^th^, 2013; the whole genome sequences of the diatoms *Thalassiosira pseudonana*, *Phaeodactylum tricornutum*, *Fragilariopsis cylindrus* and *Pseudo-nitzschia multiseries* (http://genome.jgi.doe.gov/); and publicly available genomes from *Emiliania huxleyi*, *Ectocarpus siliculosis* (http://genome.jgi.doe.gov/) and a transcriptome of *Pseudo-nitzschia granii* (http://camera.calit2.net/).

### Identification of transcriptome homologs

Translated genes encoding twelve proteins were targeted for analysis: high-affinity ferric reductase (*FRE*); multi-copper oxidase (*MCO*); iron(III) permease (*FTR*); ferritin (*FTN*); flavodoxin (*FLDA*); ferredoxin (*petF*); plastocyanin (*PCYN*); cytochrome c6 (*CYTC6*); and the Ni-, Fe-, Mn-, and Cu-Zn-superoxide dismutases (Fig 1). For each target gene, we identified reference sequences with an experimentally verified and/or a solved x-ray crystallographic protein structure (S1 Table). The reference sequences were aligned with Mafft L-INS-i [56], and trimmed on the carboxy and amino termini to match the mature protein coding regions given by the reference sequence(s) in Jalview 2.0 [57]. Hidden Markov Model (HMM) profiles of the reference sequence alignments were constructed with hmmsearch [58] and used to identify homologous sequences (based on e-value cutoff of 1e-05) in translated transcriptomic and genomic data sets. To ensure alignment quality, short fragments and extended contigs spanning less than 25% or greater than 500% of the mature protein coding regions, respectively, were removed in Jalview 2.0 [57]. Homologous sequences for *FLAV*, *FER*, *PCY*, *CYTC6* from *Thalassiosira oceanica* and *Chondrus crispus* were identified in NCBI based on annotations and for *Cu-ZnSOD*, *FeSOD*, *MnSOD*, *NiSOD*, *FRE*, *MCO*, and *FTR* translated amino acid sequences based on BLASTP (e-value cut-off <1e-05). Potential homologs were aligned to the reference sequences using Mafft FFT-NS-I [56] and trimmed on the amino and carboxy termini to match the length of the reference sequences in Jalview 2.0 [57]. Those sequences with unique insertions of over 50 amino acids were removed to minimize inclusion of mis-assemblies and to improve alignment quality. Sequences with 100% identity within individual strains were removed from the alignment.

To resolve the phylogeny of the *FTN* tree, close homologs of *FTN* from prokaryotes were identified with a BLASTP search against GenBank nr on November 8^th^, 2013 against all Bacteria, using six representative sequences as queries (*Pseudo-nitzschia multiseries* [GenBank:ACI30660.1], *Phaeodactylum tricornutum* [JGI:49895], *Goniomonas pacifica* [CAMERA:0175924348], *Fibrocapsa japonica* [CAMERA:0113935616], *Stygamoeba regulata* [CAMERA:0177662056], and *Rhizosolenia setigera* [CAMERA:0178962850]. The top scoring hits were added to the alignment of translated *FTN* sequences. RAxML was run on this alignment using the PROTGAMMAWAG model and 1,000 bootstraps to attain a higher accuracy in estimating the FTN phylogeny.

### Identification of specific functional activity by selection for known motifs

Additional identification steps were taken with the components of the reductive uptake system due to the notable divergence in sequence homology within these large gene families. Putative functions of the three elements of the reductive uptake system were assumed after filtering the aligned, translated sequences for conserved motifs of published biochemical function. For *FRE*, this warranted conservation of the FAD binding site [(H-P-F-(S/T)-(V/L/I)] and the NADPH-adenine binding motif (C-G-P) that were observed in the four *FRE* sequences from *T*. *pseudonana* and *P*. *tricornutum* [15] and initially described in yeast [12]. Valine and Alanine were permitted as replacements for Glycine in the NADPH-adenine binding motif. For *MCO*, copper-coordinating activity was assumed after filtering for conserved histidine residues within the canonical Cu-coordinating motifs described in yeast Fet3p [14]. Putative *FTR* function was assigned by filtering sequences for the “REXXE” motif implicated in iron-permeability [13].

Additionally, *NiSOD* was identified by the presence of the Ni-hook motif (H-C-X-X-P-C-G-X-Y) on the N-terminal end of the translated *NiSOD* gene, necessary for metal binding and catalysis [42]. Fe and Mn *SOD* are distinguished by conserved metal binding residues at sites 77 and 146 [39]. *FeSOD* conserves a Q at 77 and A (though G was accepted) at 146, with *MnSOD* showing a G and Q at 77 and 146, respectively.

### Phylogenetic analysis

Phylogeny was inferred with maximum-likelihood phylogenetic trees generated using RAxML version 7.6.4 (translated *FTN*) [59] or FastTree version 2.1.7 (all other translated genes) [60]. Trees were visualized in Archaeopteryx [61]. Bayesian inference was performed with MrBayes version 3.1.2 [62] using mixed amino acid rate matrices. Unless otherwise noted, consensus trees were constructed with a relative burn-in of 25% following 1,000,000 generation runs, with trees collected every 500 generations.

### Determining copy numbers of detected transcripts

Presence or absence and copy number of each gene was determined qualitatively from the phylogenetic tree for each set of translated amino acid sequences. For every strain, each translated sequence with distinct tree placements was counted as one single copy, with multiple copies given by sequences occupying unique placements on the phylogenetic tree. Sequences within the same species clustering immediately adjacent to these sequences were considered to be the same gene by assuming the closely clustered sequences to be either allelic or sequencing artifacts, though no attempts were made to distinguish between the two.

### Evolutionary relationship inferred through 18S sequence homology

A eukaryote-only SSU RNA database (version last modified June 29, 2010) of high quality 18S sequences curated and aligned by SILVA (www.arb-silva.de) was downloaded from the Mothur wiki (www.mothur.org/wiki/Silva_reference_files) on April 26, 2013 and added to a file with the 18S sequences available for all MMETSP samples and for genomes and non-MMETSP transcriptomes included in our analyses. Sequences were then re-aligned using L-INS-i option in MAFFT, empty columns removed in JalView and the tree generated with FastTree using default arguments. Diatoms all fell within a single clade; all other eukaryote branches except for select outgroups are not shown.

## Supporting Information

S1 Fig Alignment of representative diatom sequences with Yeast reference sequences. FRE1 and FRE2 from *Saccharomyces cerevisiae* aligned with translated representatives from each diatom class, illustrating conservation of FAD and NADPH-adenine binding motifs (red boxes).
(TIF)

S2 Fig Relationship of putative FRE in marine microeukaryotes and yeast. Midpoint-rooted approximately-maximum-likelihood tree of putative and known FRE amino acid sequences. Node support values are calculated from 1,000 resamples, only values over 0.5 are shown. One representative is shown from groups sharing greater than 95% similarity in unaligned sequence identity. Branches colored by organismal phylogeny: diatoms, orange; chlorophytes, green; rhodophytes, red; haptophytes and cryptophytes, purple; non-diatom stramenopiles, magenta; alveolates, blue; opisthokonts and amoebozoa, brown; excavates, pale blue; rhizaria, grey. Legend, species, and PID, from top to bottom: PtFRE1, *Phaeodactylum tricornutum*, [JGI:46928]; TpFRE1, *Thalassiosira pseudonana*, [JGI:11375]; PtFRE2, *Phaeodactylum tricornutum*, [JGI:54486]; ScFRE2, *Saccharomyces cerevisiae*, [GenBank:6322629]; ScFRE1, *Saccharomyces cerevisiae*, [GenBank:6323243]; TpFRE2, *Thalassiosira pseudonana*, [JGI:261641].
(TIF)

S3 Fig Cu-binding motifs of putative MCOs. Residues responsible for metal coordination in *Saccharomyces cerevisiae* (Taylor et al., 2005) marked with red arrows. Deviations from *S*. *cerevisiae* residues are boxed. Highlighted top rows, fungal MCOs with ferroxidase activity. Roman numerals above columns represent regions in *S*. *cerevisiae* Fet3p: I, T78 to L85; II, G121 to H128; III, H413 to H420; IV, G478 to H489.
(TIF)

S4 Fig Phylogenetic relationship of ferroxidase activity within the putative microeukaryote MCO family. Midpoint-rooted approximately-maximum-likelihood tree of putative and known MCO amino acid sequences. Node support values are calculated from 1,000 resamples, only values over 0.5 are shown. Green highlighted box indicates homologs with proposed ferroxidase activity. One representative is shown from groups sharing greater than 95% similarity in unaligned sequence identity. Branches colored by organismal phylogeny: diatoms, orange; chlorophytes, green; haptophytes and cryptophytes, purple; non-diatom stramenopiles, magenta; alveolates, blue; opisthokonts and amoebozoa, brown; excavates, pale blue; rhizaria, grey. Confidence values shown for deep branches, other omitted for clarity. Accession IDs: *Thalassiosira pseudonana* FET3, [JGI:5574]; ScFET3, *Saccharomyces cerevisiae* FET3p, [GenBank:CAA89768.1]; BaAFET3, *Blastobotrys adeninivorans*, [GenBank:CAB90817.1]; CnCNLAC1, *Cryptococcus neoformans* CNLAC1, [GenBank:EAL19727.1]; PcMCO-4, *Phanerochaete chrysosporium* MCO-4, [GenBank:AAS21672.1].
(TIF)

S5 Fig Diatoms maintain functional motifs necessary for iron permeation in *S*. *cerevisiae*. Iron permeation motifs (IPM) I and II correspond to necessary functional motifs in yeast Ftr1p (Severence et al., 2004) One representative is shown from groups sharing greater than 95% similarity in unaligned sequence identity.
(TIF)

S6 Fig Relationship of putative FTR in marine microeukaryotes and yeast. Midpoint-rooted approximately-maximum-likelihood tree of putative and known FTR amino acid sequences. Node support values are calculated from 1,000 resamples, only values over 0.5 are shown. One representative is shown from groups sharing greater than 95% similarity in unaligned sequence identity. Branches colored by organismal phylogeny: diatoms, orange; chlorophytes, green; haptophytes and cryptophytes, purple; non-diatom stramenopiles, magenta; alveolates, blue; opisthokonts and amoebozoa, brown; rhodophytes, red; rhizaria, grey. Legend, species, and accession numbers: TpFTR1, *Thalassiosira pseudonana*, [JGI:268009]; TpFTR2, [JGI:10180]; ScFTR1, *Saccharomyces cerevisiae*, [GenBank:6320993].
(TIF)

S7 Fig Diatom FTN conserves ferroxidase residues, with differences at the C terminus between two primary groups. Red arrows show ferroxidase residue sites in *Pseudo-nitzschia multiseries*. Red arrowhead marks the ambiguous positions at Glu130 and Glu131 of PmFTN, where either residue may function in ferroxidase activity. Blue arrows show conserved sites for iron release in *Rana catesbeiana*.
(TIF)

S8 Fig Midpoint-rooted approximately-maximum-likelihood tree of putative and known petF amino acid sequences. Node support values are calculated from 1,000 resamples, only values over 0.5 are shown. Gray dots indicate positions of a selection of known, chloroplast-encoded *petF*. Branches colored by organismal phylogeny: diatoms, orange; chlorophytes, green; rhodophytes, red; haptophytes and cryptophytes, purple; non-diatom stramenopiles, magenta; alveolates, blue; excavates, pale blue; rhizaria, grey. Top to bottom, accession numbers: *Cyanidioschyzon merolae*, [PDB:3AB5]; *Pyropia yezoensis*, [GenBank:YP_537001]; *Thalassiosira pseudonana*, [GenBank:YP_874492]; *Odontella sinensis*, [Swiss-Prot:P49522]; *Durinskia baltica*, [GenBank:YP_003734995]; *Thalassiosira weissflogii*, [Swiss-Prot:O98450]; *Spinacia oleracea*, [Swiss-Prot:P00224]; *Chlamydomonas reinhardtii*, [Swiss-Prot:P07839]; *Dunaliella salina*, [Swiss-Prot:P00239]; *Pisum sativum*, [Swiss-Prot:P09911]. Black dots indicate diatom sequences with known (*Thalassiosira oceanica*, [GenBank:EJK54785]) and putative transit peptides (all others). Top to bottom: *Thalassiosira* sp, MMETSP1071, [CAMERA:0181112606]; *Thalassiosira miniscula*, MMETSP0737, [CAMERA:0183726686]; *Skeletonema menzelii*, MMETSP0603, [CAMERA:0183647566]; *Thalassionema frauenfeldii*, MMETSP0786, [CAMERA:0178916612]; *Grammatophora oceanica*, MMETSP0009 [CAMPEP:0194032050]; *Thalassiosira oceanica*, [GenBank:EJK54785]; *Skeletonema costatum*, MMETSP0013, [CAMERA:0113387486]; *Skeletonema marinoi*, MMETSP0320, [CAMERA:0115946752]; *Minutocellus polymorphus*, MMETSP1070, [CAMERA:0181038080]; *Odontella aurita*, MMETSP0015, [CAMERA:0113537566]; *Thalassiosira miniscula*, MMETSP0737, [CAMERA:0183720344]; *Thalassionema frauenfeldii*, MMETSP0786, [CAMERA:0178915392].
(TIF)

S9 Fig Approximate maximum-likelihood tree of flavodoxin transcripts. Midpoint-rooted approximately-maximum-likelihood tree of putative and known flavodoxin amino acid sequences. Node support values are calculated from 1,000 resamples, only values over 0.5 are shown. Branch labels hidden for clarity. Branches colored by organismal phylogeny: diatoms, orange; chlorophytes, green; rhodophytes, red; haptophytes and cryptophytes, lavender; non-diatom stramenopiles, magenta; alveolates, blue; excavates, pale blue; rhizaria, gray. Accession numbers for reference sequences: TpFLAV-I, *Thalassiosira pseudonana* [JGI:19141]; TwFLAV-II, *Thalassiosira weissflogii* MMETSP0879 [CAMERA:0171358606]. Accession numbers for species with two or more copies of clade II FLAV: PiFLAV-IIa, *Proboscia inermis* MMETSP0816 [CAMERA:0171295654]; PiFLAV-IIb, *Proboscia inermis* MMETSP0816 [CAMERA:0171313668]; PifLAV-IIc, *Proboscia inermis* MMETSP0816 [CAMERA:0171319130], *Proboscia inermis* MMETSP0816 [CAMERA:0171292874] and *Proboscia inermis* MMETSP0816 [CAMERA:0171292998]; SrFLAV-IIa, *Synedropsis recta* MMETSP1176 [CAMERA:0119013084]; SrFLAV-IIb, *Synedropsis recta* MMETSP1176 [CAMERA:0119018232]; FkFLAV-IIa, *Fragilariopsis kerguelensis* MMETSP0735 [CAMERA:0170933076], *Fragilariopsis kerguelensis* MMETSP0733 [CAMERA:0170785742], and *Fragilariopsis kerguelensis* MMETSP0735 [CAMERA:0170936014]; FkFLAV-IIb, *Fragilariopsis kerguelensis* MMETSP0736 [CAMERA:0171020946], *Fragilariopsis kerguelensis* MMETSP0735 [CAMERA:0170914250], *Fragilariopsis kerguelensis* MMETSP0736 [CAMERA:0170997016], and *Fragilariopsis kerguelensis* MMETSP0735 [CAMERA:0170928162].
(TIF)

S10 Fig Alignment of known and putative PCYN sequences. Cu-coordinating residues from *P*. *nigra* indicated with red arrows. Alignment starts from G51 of *P*. *nigra* plastocyanin *a*. Starting from top, ID and accession numbers: *Populus nigra*, [GenBank:CAA90564.1]; *Populus nigra*, [GenBank:1402239A]; *Thalassiosira oceanica*, [Swiss-Prot:D2Z0I2]; *Fragilariopsis cylindrus* [JGI:272258]; *Pseudo-nitzschia granii* deg7180000014200 frame0; *Corethron hystrix*, MMETSP0010, [CAMERA:0113306274]; *Coscinodiscus wailesii*, MMETSP1066, [CAMERA:0172483904]; *Ditylum brightwellii*, MMETSP1063, [CAMERA:0180970060]; *Fragilariopsis kerguelensis*, MMETSP0733, [CAMERA:0170771410]; *Fragilariopsis kerguelensis*, MMETSP0733, [CAMERA:0170793268]; *Fragilariopsis kerguelensis*, MMETSP0734, [CAMERA:0170902168]; *Proboscia inermis*, MMETSP0816, [CAMERA:0171306160]; *Pseudo-nitzschia arenysensis*, MMETSP0329, [CAMERA:0116141514]; *Pseudo-nitzschia heimii*, MMETSP1423, [CAMPEP:0197183406]; *Rhizosolenia setigera*, MMETSP0789, [CAMERA:0178972290]; *Striatella unipunctata*, MMETSP0800, [CAMERA:0118690216]; *Fragilariopsis kerguelensis*, MMETSP0734, [CAMERA:0170889260]; *Pseudo-nitzschia heimii*, MMETSP1423, [CAMPEP:0197180752]; *Pseudo-nitzschia heimii*, MMETSP1423, [CAMPEP:0197182106].
(TIF)

S11 Fig Alignment of Fe and Mn SOD sequences from representative diatoms. Metal binding residues differentiating Fe and Mn SOD activity marked with red boxes. Putative FeSOD accession numbers: *Asterionellopsis glacialis*, MMETSP1394, [CAMPEP:0197135456]; *Odontella sinensis*, MMETSP0160, [CAMERA:0183305568]; *Extubocellulus spinifer*, MMETSP0696, [CAMERA:0178491238]; *Ditylum brightwellii*, MMETSP1062, [CAMERA:0180922322]; *Chaetoceros* cf, MMETSP1336, [CAMERA:0119456406]; *Skeletonema costatum*, MMETSP0013, [CAMERA:0113409648]; *Thalassiosira oceanica*, [GenBank:EJK73388]; *Thalassiosira pseudonana*, [JGI:263062]; *Thalassiosira punctigera*, MMETSP1067, [CAMERA:0172528302]; *Detonula confervacea*, MMETSP1058, [CAMERA:0172326082]; *Stephanopyxis turris*, MMETSP0794, [CAMPEP:0195508046]. Putative MnSODs accession numbers: *Amphora coffeaeformis*, MMETSP0316, [CAMERA:0170658046]; *Phaeodactylum tricornutum*, [JGI:42832]; *Nitzschia* sp, MMETSP0014, [CAMERA:0113451442]; *Fragilariopsis kerguelensis*, MMETSP0735, [CAMERA:0170940508]; *Pseudo-nitzschia arenysensis*, MMETSP0329, [CAMERA:0116142848]; *Asterionellopsis glacialis*, MMETSP1394, [CAMPEP:0197142074]; *Thalassionema frauenfeldii*, MMETSP0786, [CAMERA:0178920282]; *Nanofrustulum* sp, MMETSP1361, [CAMPEP:0202481740]; *Odontella aurita*, MMETSP0015, [CAMERA:0113546944]; *Minutocellus polymorphus*, MMETSP1070, [CAMERA:0181044600]; *Ditylum brightwellii*, MMETSP1062, [CAMERA:0180939824]; *Skeletonema costatum*, MMETSP0013, [CAMERA:0113403410]; *Cyclotella meneghiniana*, MMETSP1057, [CAMERA:0172267766]; *Detonula confervacea*, MMETSP1058, [CAMERA:0172309720]; *Chaetoceros* sp, MMETSP0200, [CAMERA:0176485652]; *Stephanopyxis turris*, MMETSP0794, [CAMPEP:0195522388]; *Rhizosolenia setigera*, MMETSP0789, [CAMERA:0178949314]; *Leptocylindrus* CCMP1586, MMETSP1362, [CAMPEP:0196811398].
(TIF)

S12 Fig Alignment of known CuZnSOD sequences with a representative diatom selection. N-terminus sequence trimmed to aligned region at position 14 of *S*. *oleracea*. Reference sequences (top), diatom sequences that maintain all binding residues (middle) and other diatom homologs (bottom), separated by horizontal black lines. Key Cu and Zn binding residues marked with red arrows. MMETSP and accession IDs: *Spinacia oleracea*, [PDB:1SRD]; *Saccharomyces cerevisiae*, [PDB:1SDY]; *Rhizosolenia setigera*, MMETSP0789, [CAMERA:0178950748]; *Stephanopyxis turris*, MMETSP0794, [CAMPEP:0195542640]; *Stephanopyxis turris*, MMETSP0794, [CAMPEP:0195519432]; *Nitzschia punctata*, MMETSP0747, [CAMERA:0178859982]; *Nanofrustulum* sp, MMETSP1361, [CAMPEP:0202480120]; *Rhizosolenia setigera*, MMETSP0789, [CAMERA:0178941642]; *Stephanopyxis turris*, MMETSP0794, [CAMPEP:0195524364]; *Thalassiosira weissflogii*, MMETSP0879, [CAMERA:0171359706]; *Thalassionema frauenfeldii*, MMETSP0786, [CAMERA:0178894792]; *Grammatophora oceanica*, MMETSP0009, [CAMPEP:0194033848]; *Synedropsis recta*, MMETSP1176, [CAMERA:0119013068]; *Amphiprora* sp, MMETSP0724, [CAMERA:0168741714]; *Amphiprora paludosa*, MMETSP1065, [CAMERA:0172471802]; *Amphora coffeaeformis*, MMETSP0316, [CAMERA:0170661724]; *Phaeodactylum tricornutum*, [JGI:15852]; *Pseudo-nitzschia multiseries*, [JGI:220645]; *Fragilariopsis kerguelensis*, MMETSP0735, [CAMERA:0170910884]; *Fragilariopsis cylindrus* [JGI:269494]; *Nitzschia punctata*, MMETSP0747, [CAMERA:0178882228]; *Nitzschia* sp, MMETSP0014, [CAMERA:0113522044]; *Cylindrotheca closterium*, MMETSP0017, [CAMERA:0113603408]; *Stauroneis constricta*, MMETSP1352, [CAMERA:0119562388]; *Nanofrustulum* sp, MMETSP1361, [CAMPEP:0202482534]; *Striatella unipunctata*, MMETSP0800, [CAMERA:0118702026]; *Extubocellulus spinifer*, MMETSP0698, [CAMERA:0178620380]; *Minutocellus polymorphus*, MMETSP1070, [CAMERA:0181043470]; *Proboscia inermis*, MMETSP0816, [CAMERA:0171322060]; *Aulacoseira subarctica*, MMETSP1064, [CAMERA:0172423284]; *Rhizosolenia setigera*, MMETSP0789, [CAMERA:0178948996]; *Odontella sinensis*, MMETSP0160, [CAMERA:0183307194]; *Odontella aurita*, MMETSP0015, [CAMERA:0113536162]; *Ditylum brightwellii*, MMETSP1063, [CAMERA:0180987508]; *Coscinodiscus wailesii*, MMETSP1066, [CAMERA:0172489328]; *Stephanopyxis turris*, MMETSP0794, [CAMPEP:0195518104].
(TIF)

S13 Fig Relationship of putative eukaryotic NiSOD showing placement of UBQ-NiSOD fusion proteins. Midpoint-rooted approximately-maximum-likelihood tree of putative NiSOD amino acid sequences. Node support values are calculated from 1,000 resamples, only values over 0.5 are shown. One representative is shown from groups sharing greater than 95% similarity in aligned sequence identity. Sequences with an UBQ-coding region on the N-terminus are indicated by red squares. UBQ and other residues preceding the Ni-hook were trimmed from NiSOD-coding region prior to phylogenetic analyses to reflect relationship of NiSOD only. Branches colored by organismal phylogeny: diatoms, orange; chlorophytes, green; haptophytes and cryptophytes, lavender; non-diatom stramenopiles, magenta; alveolates, blue; unikonts, brown; excavates, pale blue; rhizaria, gray.
(TIF)

S1 Table Reference sequences used for HMM profile. For each gene investigated, this table lists the organism, source repository, accession #, experimental support if any, and PDB structure accession number if available.
(XLSX)
