# Supplementary figures and images for: Diversity and Evolutionary History of Iron Metabolism Genes in Diatoms
Source: PLoS One. 2015 Jun 8;10(6):e0129081. doi: 10.1371/journal.pone.0129081 (PMC4460010; doi:10.1371/journal.pone.0129081)

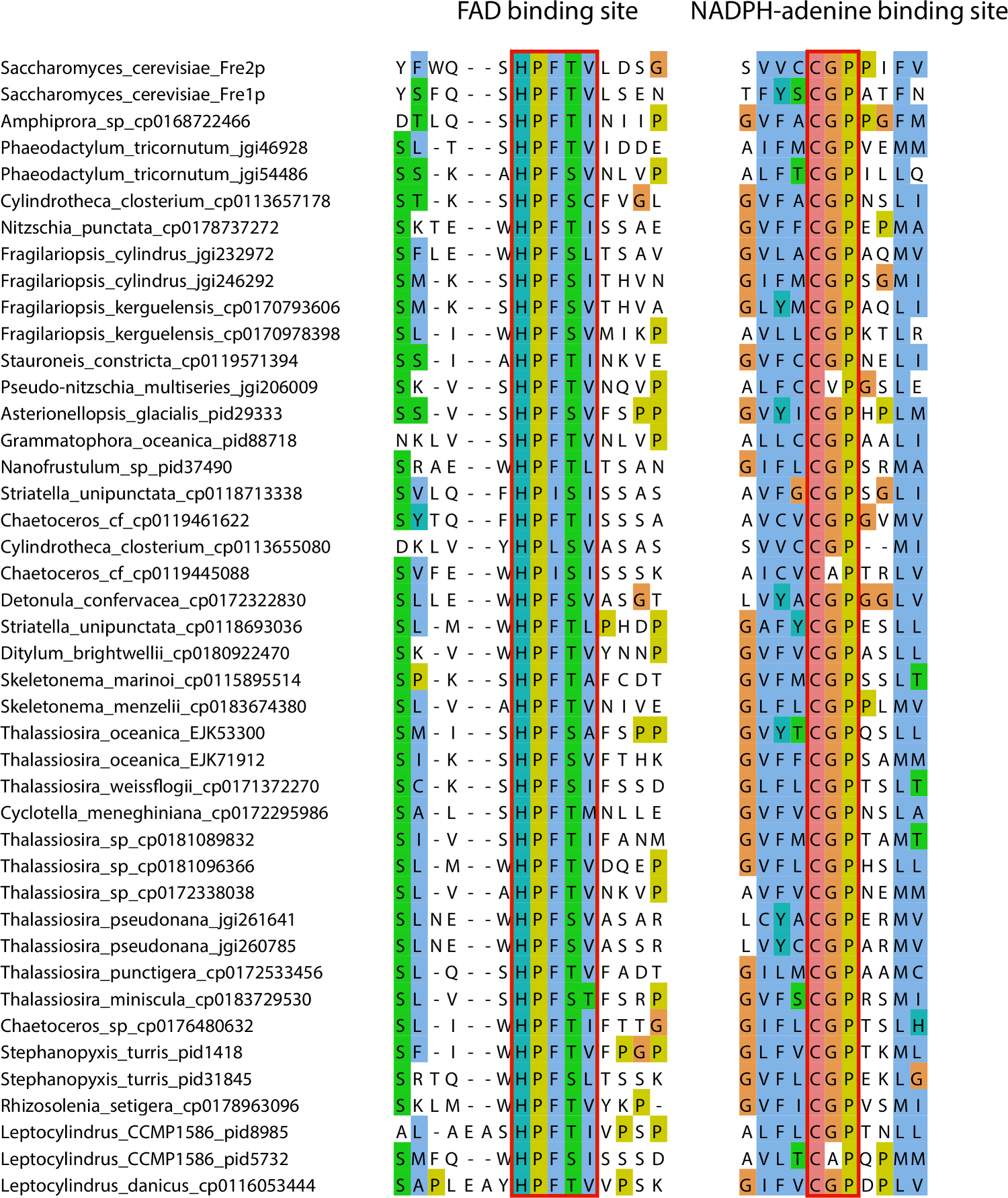

Supplement: S1 Fig — FRE1 and FRE2 from Saccharomyces cerevisiae aligned with translated representatives from each diatom class, illustrating conservation of FAD and NADPH-adenine binding motifs (red boxes). (TIF) [file pone.0129081.s001.tif]

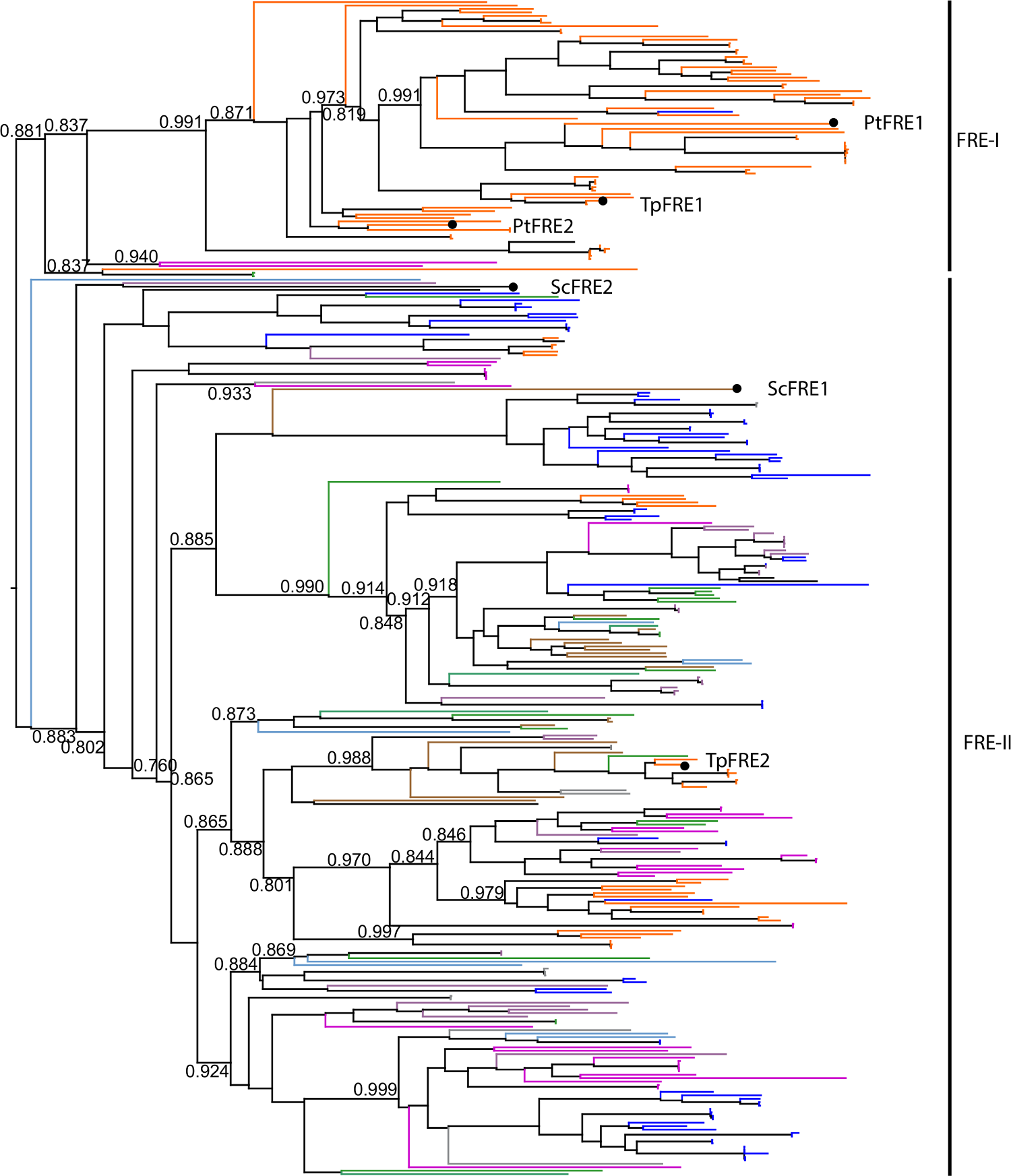

Supplement: S2 Fig — Midpoint-rooted approximately-maximum-likelihood tree of putative and known FRE amino acid sequences. Node support values are calculated from 1,000 resamples, only values over 0.5 are shown. One representative is shown from groups sharing greater than 95% similarity in unaligned sequence identity. Branches colored by organismal phylogeny: diatoms, orange; chlorophytes, green; rhodophytes, red; haptophytes and cryptophytes, purple; non-diatom stramenopiles, magenta; alveolates, blue; opisthokonts and amoebozoa, brown; excavates, pale blue; rhizaria, grey. Legend, species, and PID, from top to bottom: PtFRE1, Phaeodactylum tricornutum, [JGI:46928]; TpFRE1, Thalassiosira pseudonana, [JGI:11375]; PtFRE2, Phaeodactylum tricornutum, [JGI:54486]; ScFRE2, Saccharomyces cerevisiae, [GenBank:6322629]; ScFRE1, Saccharomyces cerevisiae, [GenBank:6323243]; TpFRE2, Thalassiosira pseudonana, [JGI:261641]. (TIF) [file pone.0129081.s002.tif]

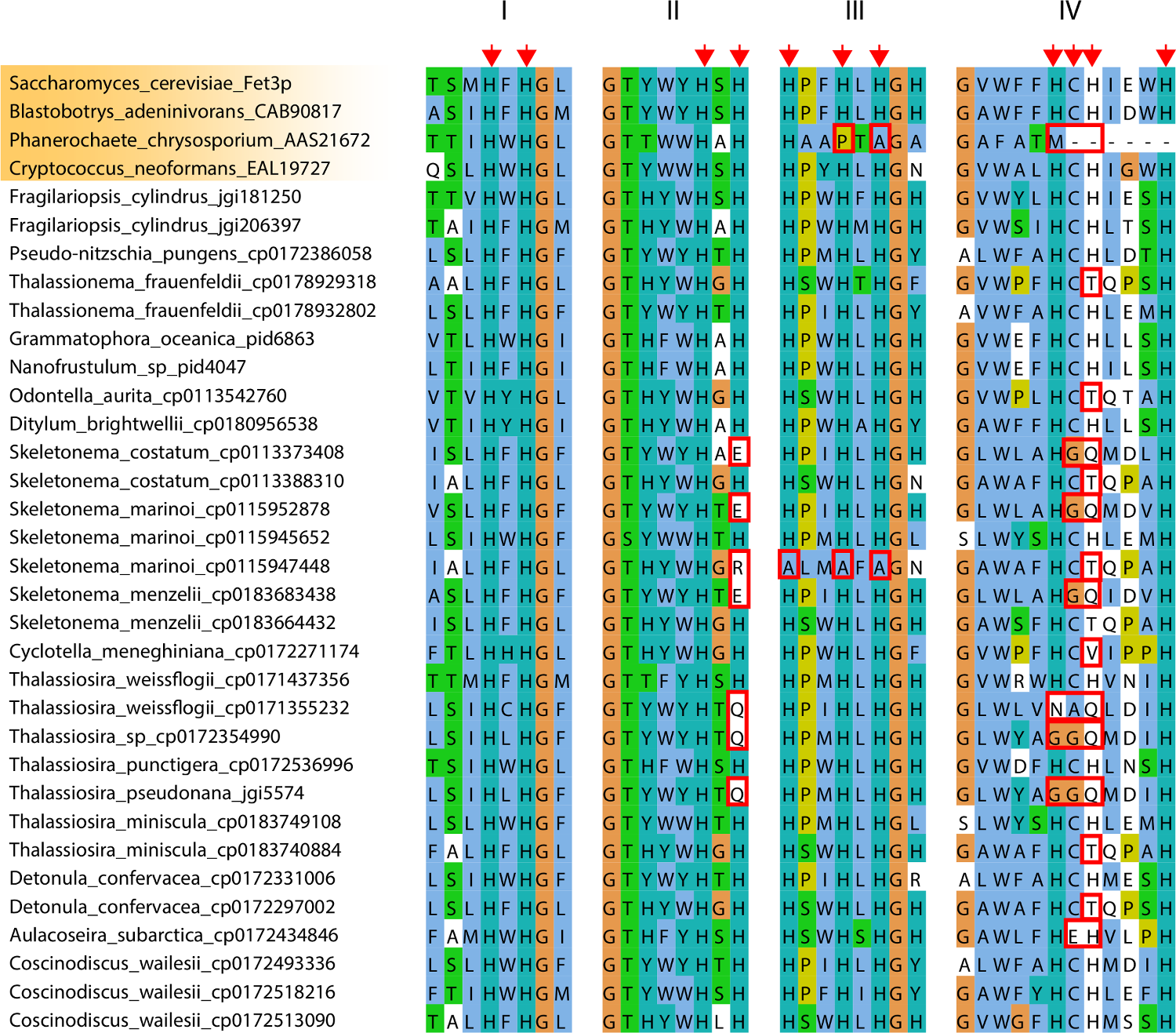

Supplement: S3 Fig — Residues responsible for metal coordination in Saccharomyces cerevisiae (Taylor et al., 2005) marked with red arrows. Deviations from S. cerevisiae residues are boxed. Highlighted top rows, fungal MCOs with ferroxidase activity. Roman numerals above columns represent regions in S. cerevisiae Fet3p: I, T78 to L85; II, G121 to H128; III, H413 to H420; IV, G478 to H489. (TIF) [file pone.0129081.s003.tif]

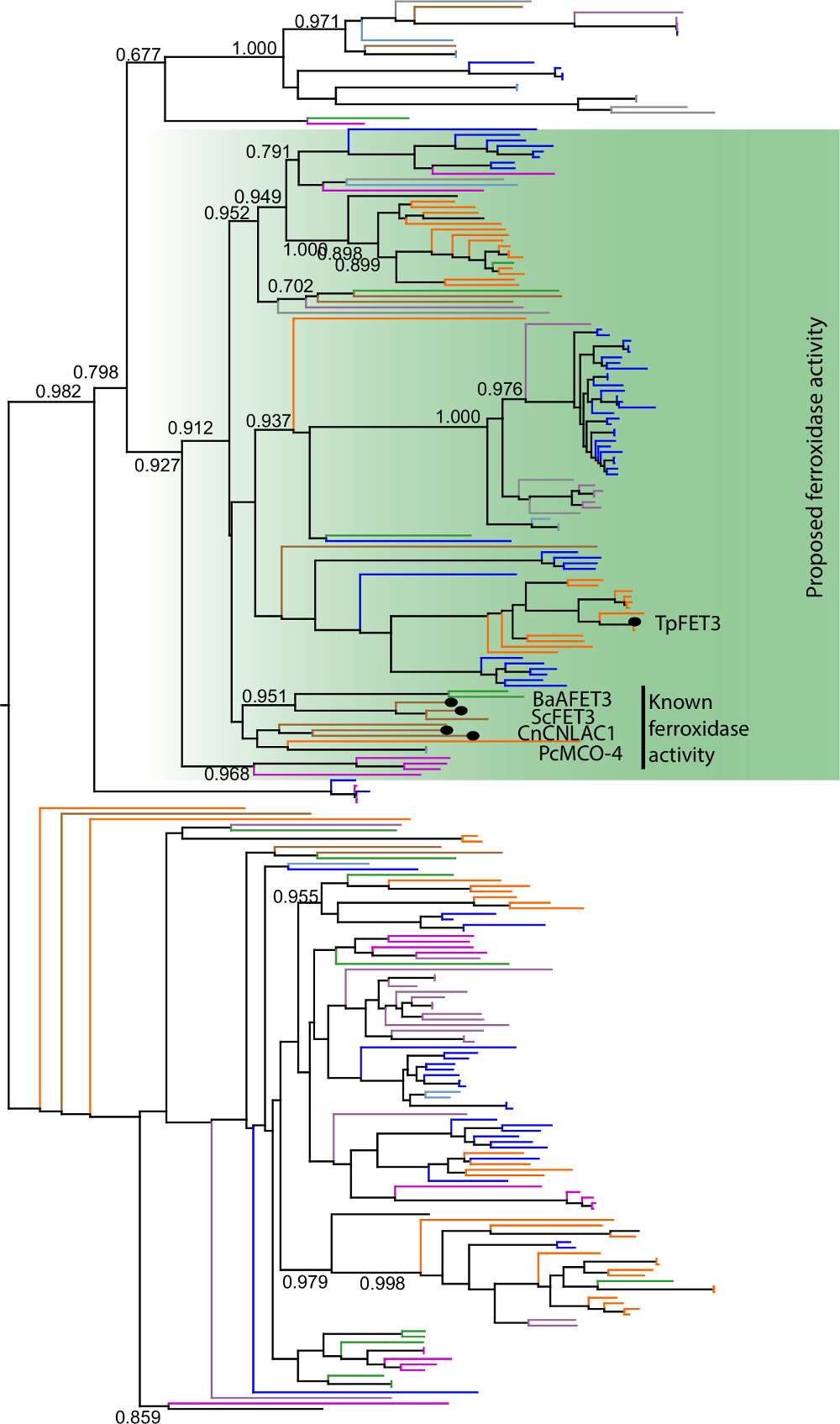

Supplement: S4 Fig — Midpoint-rooted approximately-maximum-likelihood tree of putative and known MCO amino acid sequences. Node support values are calculated from 1,000 resamples, only values over 0.5 are shown. Green highlighted box indicates homologs with proposed ferroxidase activity. One representative is shown from groups sharing greater than 95% similarity in unaligned sequence identity. Branches colored by organismal phylogeny: diatoms, orange; chlorophytes, green; haptophytes and cryptophytes, purple; non-diatom stramenopiles, magenta; alveolates, blue; opisthokonts and amoebozoa, brown; excavates, pale blue; rhizaria, grey. Confidence values shown for deep branches, other omitted for clarity. Accession IDs: Thalassiosira pseudonana FET3, [JGI:5574]; ScFET3, Saccharomyces cerevisiae FET3p, [GenBank:CAA89768.1]; BaAFET3, Blastobotrys adeninivorans, [GenBank:CAB90817.1]; CnCNLAC1, Cryptococcus neoformans CNLAC1, [GenBank:EAL19727.1]; PcMCO-4, Phanerochaete chrysosporium MCO-4, [GenBank:AAS21672.1]. (TIF) [file pone.0129081.s004.tif]

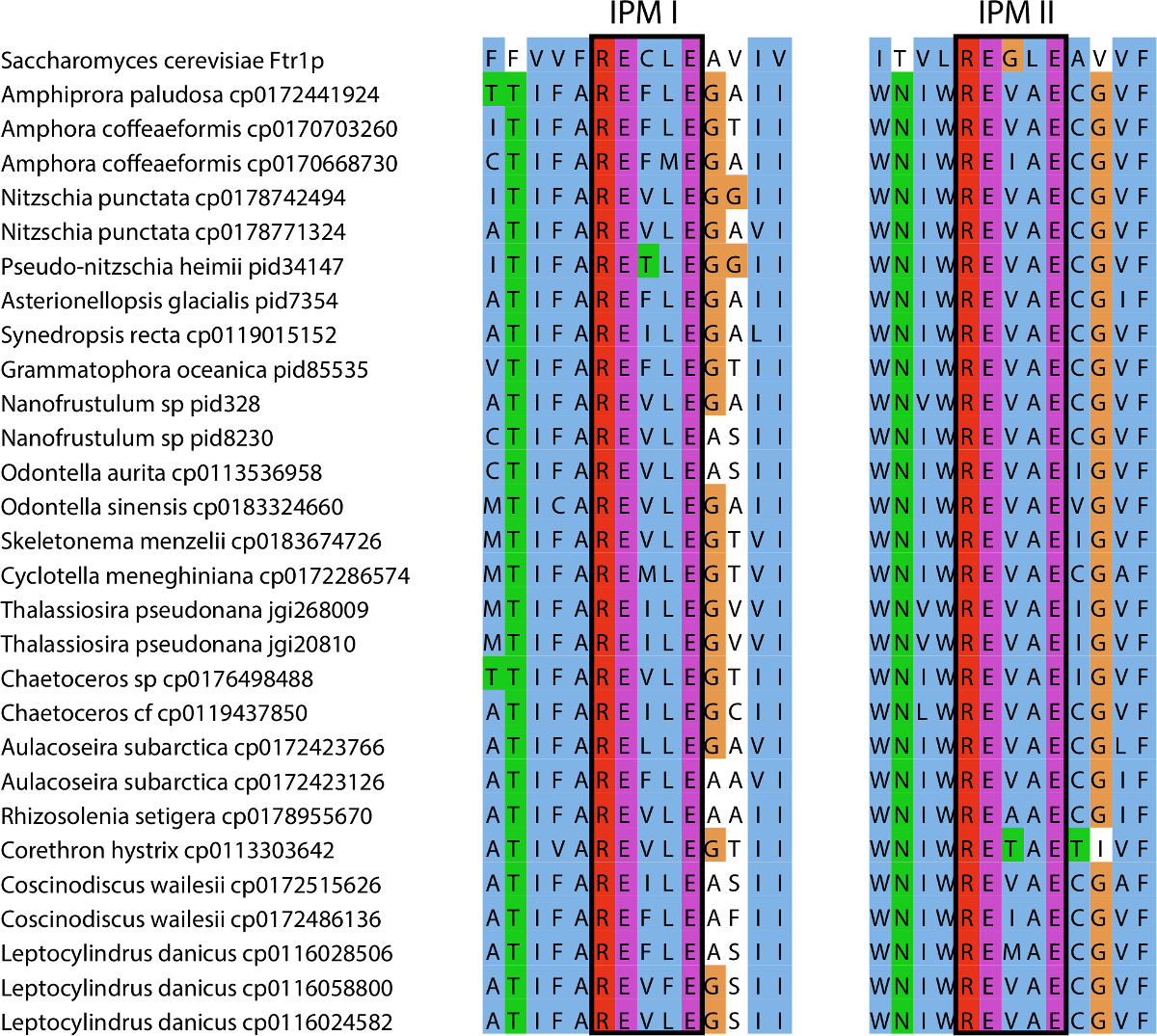

Supplement: S5 Fig — Iron permeation motifs (IPM) I and II correspond to necessary functional motifs in yeast Ftr1p (Severence et al., 2004) One representative is shown from groups sharing greater than 95% similarity in unaligned sequence identity. (TIF) [file pone.0129081.s005.tif]

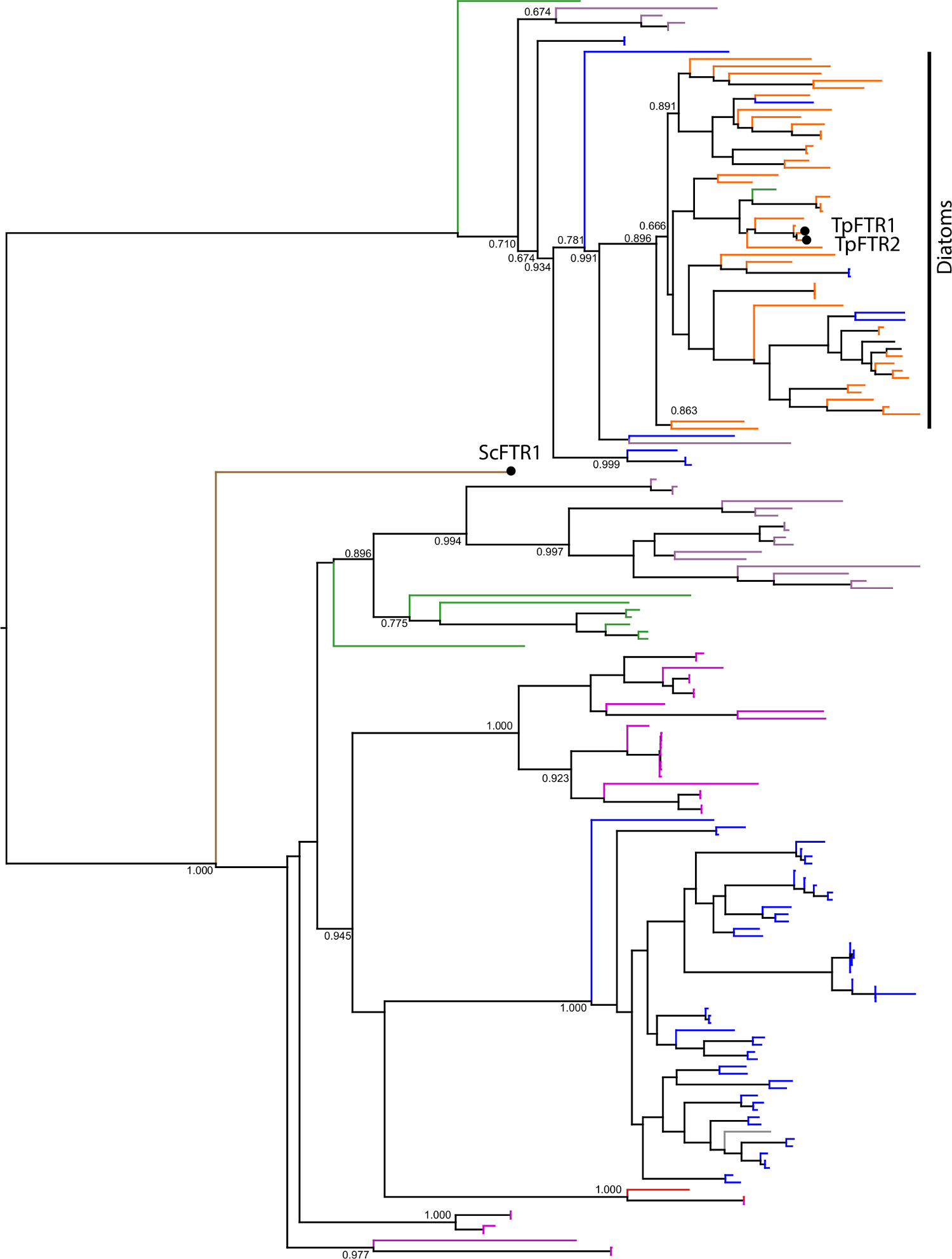

Supplement: S6 Fig — Midpoint-rooted approximately-maximum-likelihood tree of putative and known FTR amino acid sequences. Node support values are calculated from 1,000 resamples, only values over 0.5 are shown. One representative is shown from groups sharing greater than 95% similarity in unaligned sequence identity. Branches colored by organismal phylogeny: diatoms, orange; chlorophytes, green; haptophytes and cryptophytes, purple; non-diatom stramenopiles, magenta; alveolates, blue; opisthokonts and amoebozoa, brown; rhodophytes, red; rhizaria, grey. Legend, species, and accession numbers: TpFTR1, Thalassiosira pseudonana, [JGI:268009]; TpFTR2, [JGI:10180]; ScFTR1, Saccharomyces cerevisiae, [GenBank:6320993]. (TIF) [file pone.0129081.s006.tif]

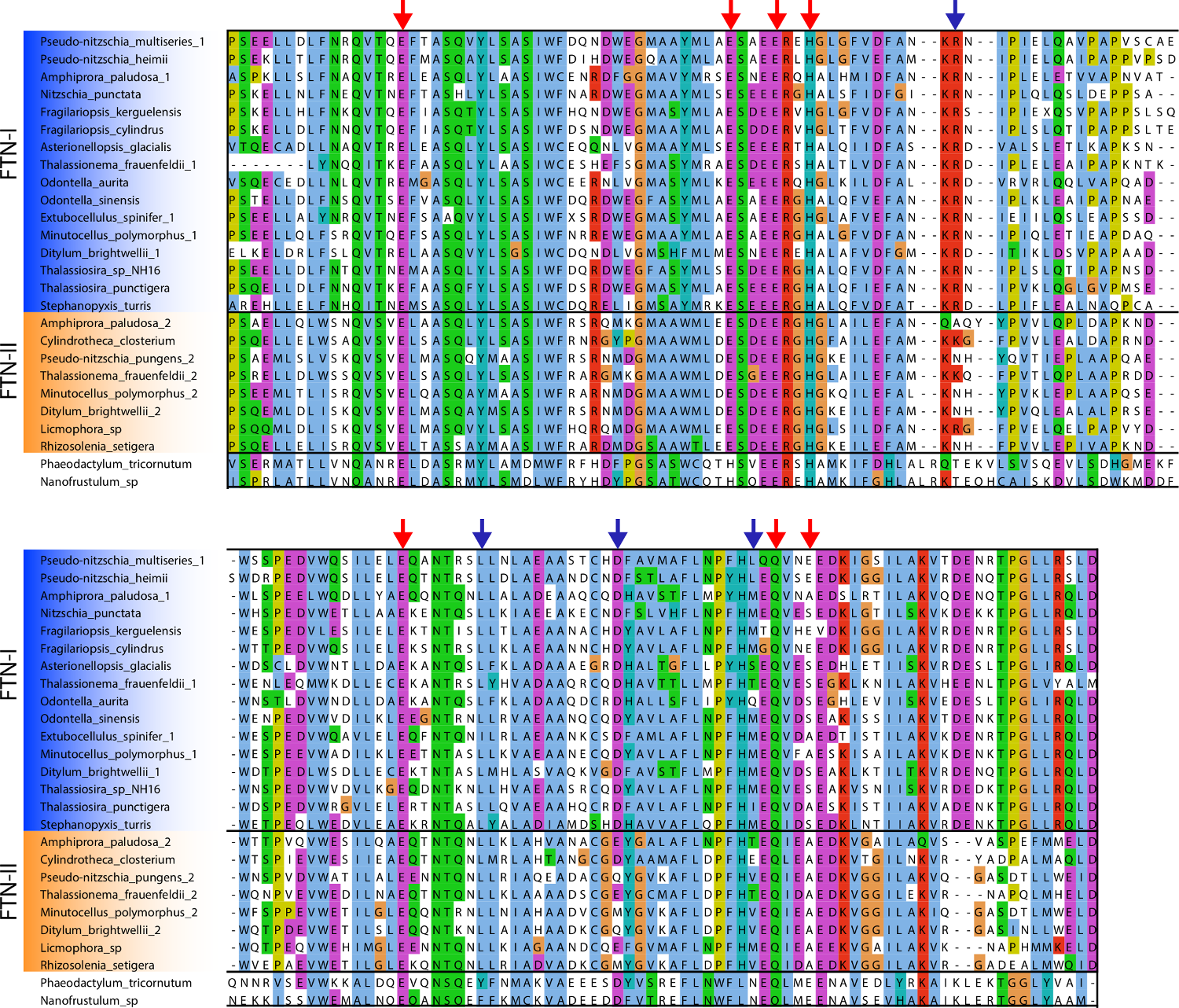

Supplement: S7 Fig — Red arrows show ferroxidase residue sites in Pseudo-nitzschia multiseries. Red arrowhead marks the ambiguous positions at Glu130 and Glu131 of PmFTN, where either residue may function in ferroxidase activity. Blue arrows show conserved sites for iron release in Rana catesbeiana. (TIF) [file pone.0129081.s007.tif]

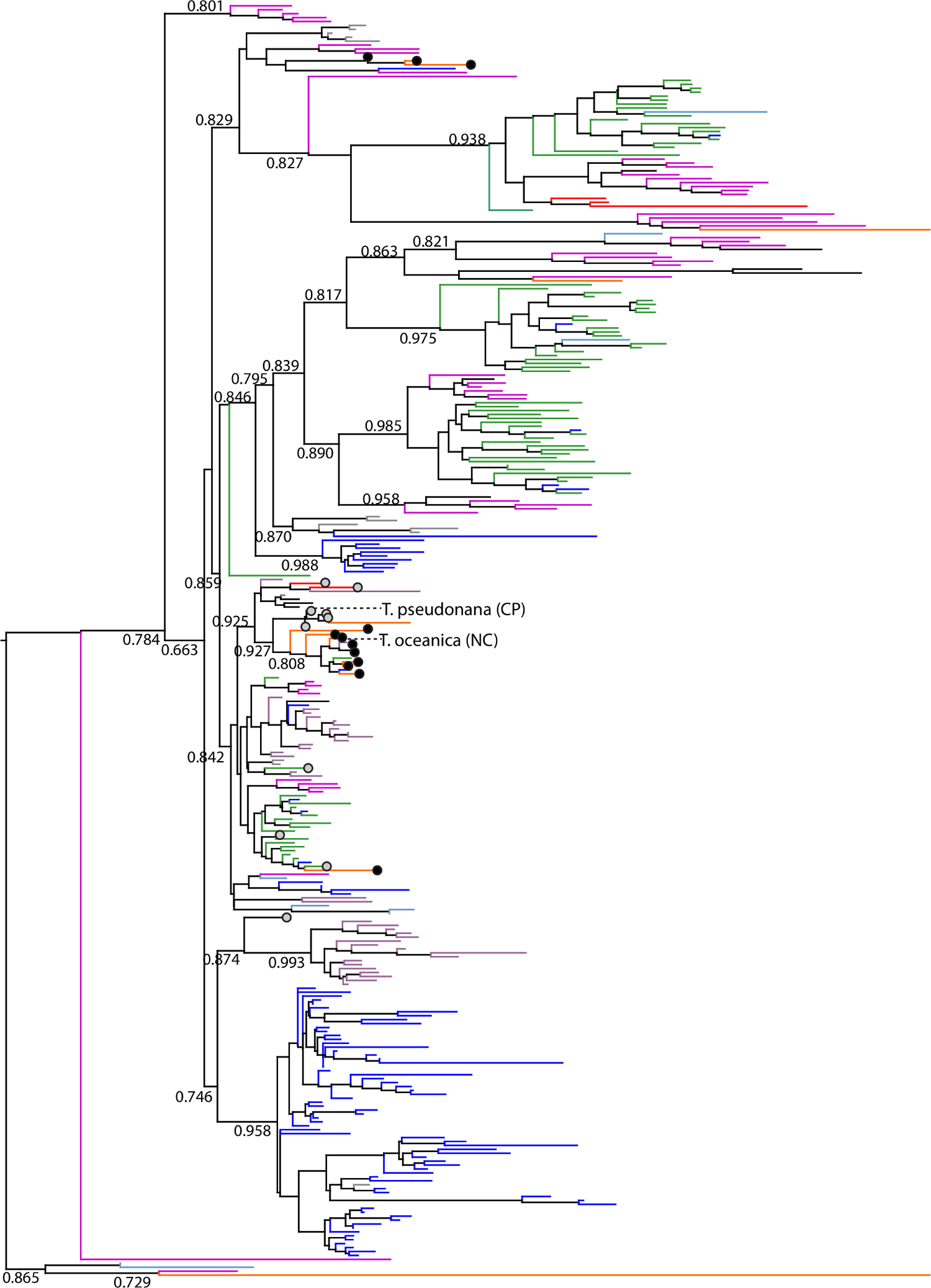

Supplement: S8 Fig — Node support values are calculated from 1,000 resamples, only values over 0.5 are shown. Gray dots indicate positions of a selection of known, chloroplast-encoded petF. Branches colored by organismal phylogeny: diatoms, orange; chlorophytes, green; rhodophytes, red; haptophytes and cryptophytes, purple; non-diatom stramenopiles, magenta; alveolates, blue; excavates, pale blue; rhizaria, grey. Top to bottom, accession numbers: Cyanidioschyzon merolae, [PDB:3AB5]; Pyropia yezoensis, [GenBank:YP_537001]; Thalassiosira pseudonana, [GenBank:YP_874492]; Odontella sinensis, [Swiss-Prot:P49522]; Durinskia baltica, [GenBank:YP_003734995]; Thalassiosira weissflogii, [Swiss-Prot:O98450]; Spinacia oleracea, [Swiss-Prot:P00224]; Chlamydomonas reinhardtii, [Swiss-Prot:P07839]; Dunaliella salina, [Swiss-Prot:P00239]; Pisum sativum, [Swiss-Prot:P09911]. Black dots indicate diatom sequences with known (Thalassiosira oceanica, [GenBank:EJK54785]) and putative transit peptides (all others). Top to bottom: Thalassiosira sp, MMETSP1071, [CAMERA:0181112606]; Thalassiosira miniscula, MMETSP0737, [CAMERA:0183726686]; Skeletonema menzelii, MMETSP0603, [CAMERA:0183647566]; Thalassionema frauenfeldii, MMETSP0786, [CAMERA:0178916612]; Grammatophora oceanica, MMETSP0009 [CAMPEP:0194032050]; Thalassiosira oceanica, [GenBank:EJK54785]; Skeletonema costatum, MMETSP0013, [CAMERA:0113387486]; Skeletonema marinoi, MMETSP0320, [CAMERA:0115946752]; Minutocellus polymorphus, MMETSP1070, [CAMERA:0181038080]; Odontella aurita, MMETSP0015, [CAMERA:0113537566]; Thalassiosira miniscula, MMETSP0737, [CAMERA:0183720344]; Thalassionema frauenfeldii, MMETSP0786, [CAMERA:0178915392]. (TIF) [file pone.0129081.s008.tif]

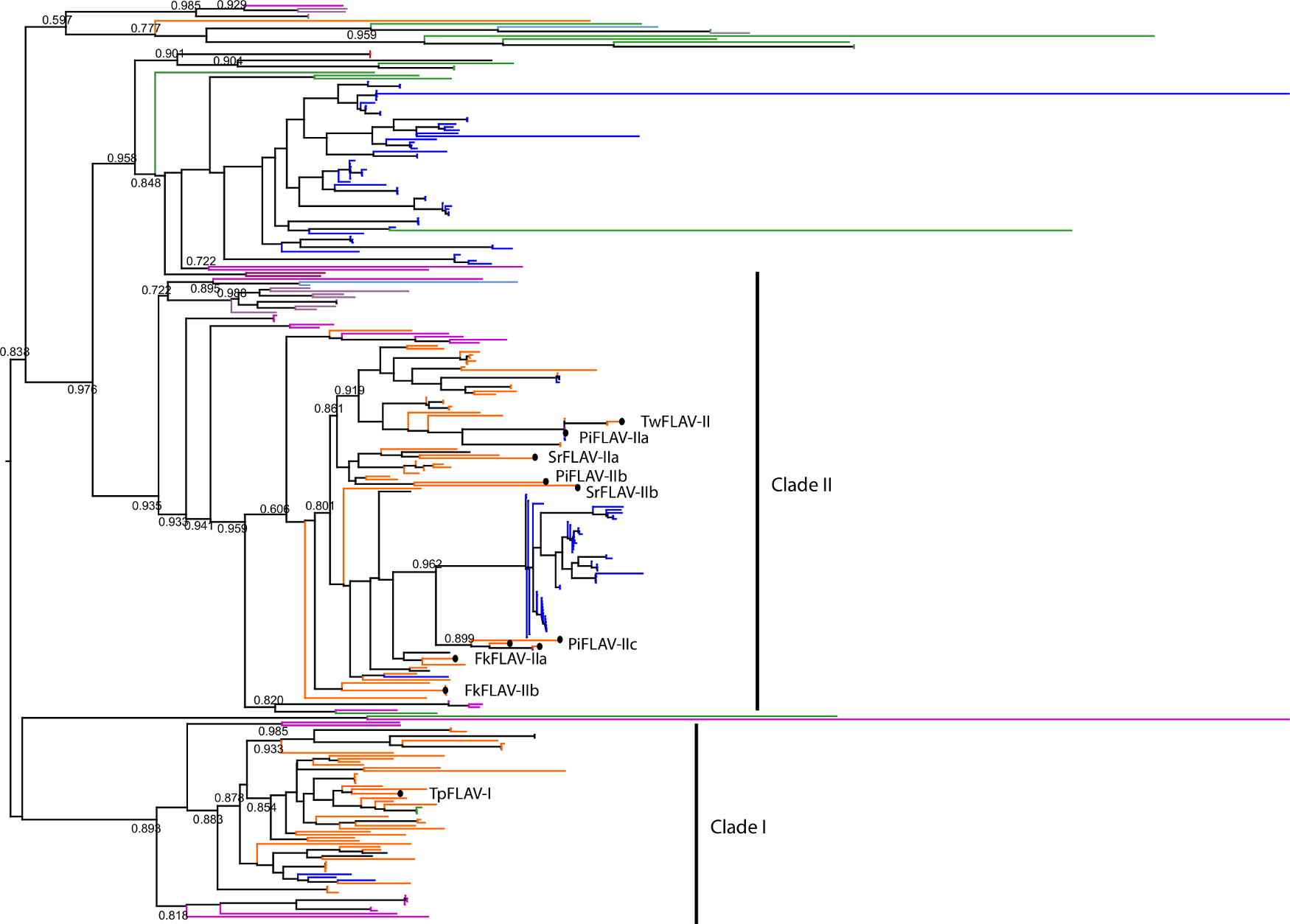

Supplement: S9 Fig — Midpoint-rooted approximately-maximum-likelihood tree of putative and known flavodoxin amino acid sequences. Node support values are calculated from 1,000 resamples, only values over 0.5 are shown. Branch labels hidden for clarity. Branches colored by organismal phylogeny: diatoms, orange; chlorophytes, green; rhodophytes, red; haptophytes and cryptophytes, lavender; non-diatom stramenopiles, magenta; alveolates, blue; excavates, pale blue; rhizaria, gray. Accession numbers for reference sequences: TpFLAV-I, Thalassiosira pseudonana [JGI:19141]; TwFLAV-II, Thalassiosira weissflogii MMETSP0879 [CAMERA:0171358606]. Accession numbers for species with two or more copies of clade II FLAV: PiFLAV-IIa, Proboscia inermis MMETSP0816 [CAMERA:0171295654]; PiFLAV-IIb, Proboscia inermis MMETSP0816 [CAMERA:0171313668]; PifLAV-IIc, Proboscia inermis MMETSP0816 [CAMERA:0171319130], Proboscia inermis MMETSP0816 [CAMERA:0171292874] and Proboscia inermis MMETSP0816 [CAMERA:0171292998]; SrFLAV-IIa, Synedropsis recta MMETSP1176 [CAMERA:0119013084]; SrFLAV-IIb, Synedropsis recta MMETSP1176 [CAMERA:0119018232]; FkFLAV-IIa, Fragilariopsis kerguelensis MMETSP0735 [CAMERA:0170933076], Fragilariopsis kerguelensis MMETSP0733 [CAMERA:0170785742], and Fragilariopsis kerguelensis MMETSP0735 [CAMERA:0170936014]; FkFLAV-IIb, Fragilariopsis kerguelensis MMETSP0736 [CAMERA:0171020946], Fragilariopsis kerguelensis MMETSP0735 [CAMERA:0170914250], Fragilariopsis kerguelensis MMETSP0736 [CAMERA:0170997016], and Fragilariopsis kerguelensis MMETSP0735 [CAMERA:0170928162]. (TIF) [file pone.0129081.s009.tif]

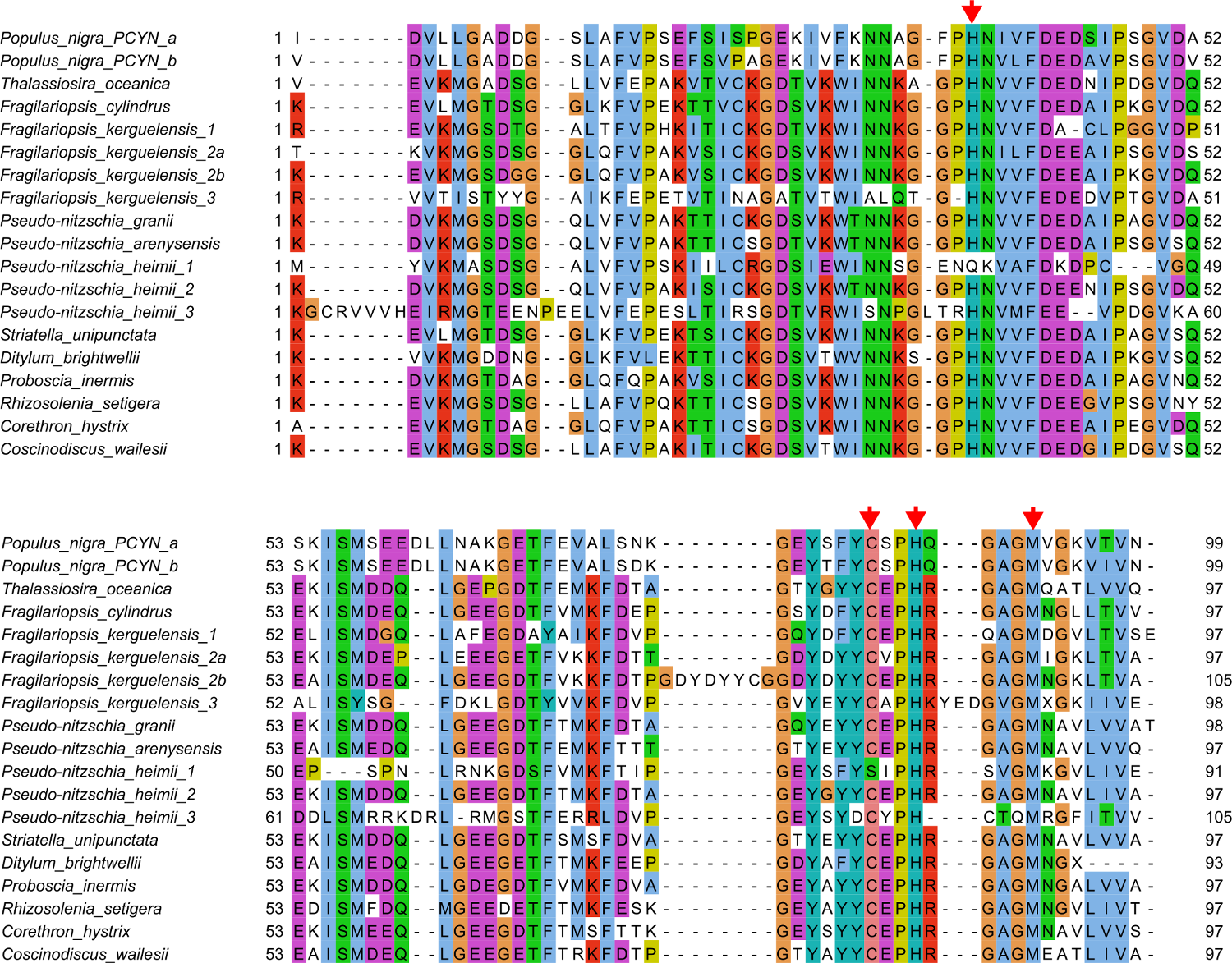

Supplement: S10 Fig — Cu-coordinating residues from P. nigra indicated with red arrows. Alignment starts from G51 of P. nigra plastocyanin a. Starting from top, ID and accession numbers: Populus nigra, [GenBank:CAA90564.1]; Populus nigra, [GenBank:1402239A]; Thalassiosira oceanica, [Swiss-Prot:D2Z0I2]; Fragilariopsis cylindrus [JGI:272258]; Pseudo-nitzschia granii deg7180000014200 frame0; Corethron hystrix, MMETSP0010, [CAMERA:0113306274]; Coscinodiscus wailesii, MMETSP1066, [CAMERA:0172483904]; Ditylum brightwellii, MMETSP1063, [CAMERA:0180970060]; Fragilariopsis kerguelensis, MMETSP0733, [CAMERA:0170771410]; Fragilariopsis kerguelensis, MMETSP0733, [CAMERA:0170793268]; Fragilariopsis kerguelensis, MMETSP0734, [CAMERA:0170902168]; Proboscia inermis, MMETSP0816, [CAMERA:0171306160]; Pseudo-nitzschia arenysensis, MMETSP0329, [CAMERA:0116141514]; Pseudo-nitzschia heimii, MMETSP1423, [CAMPEP:0197183406]; Rhizosolenia setigera, MMETSP0789, [CAMERA:0178972290]; Striatella unipunctata, MMETSP0800, [CAMERA:0118690216]; Fragilariopsis kerguelensis, MMETSP0734, [CAMERA:0170889260]; Pseudo-nitzschia heimii, MMETSP1423, [CAMPEP:0197180752]; Pseudo-nitzschia heimii, MMETSP1423, [CAMPEP:0197182106]. (TIF) [file pone.0129081.s010.tif]

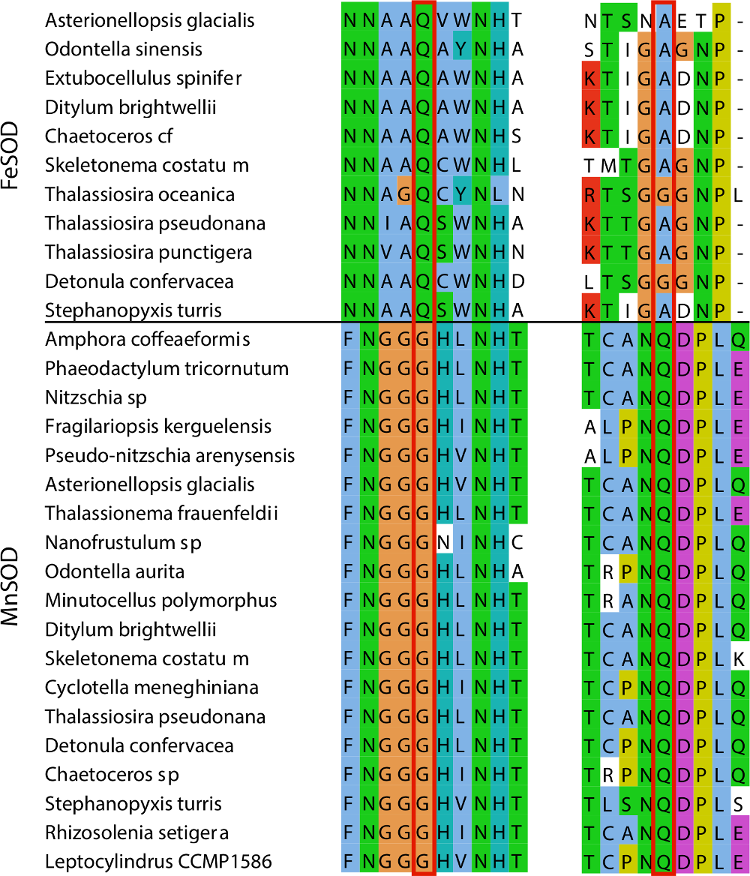

Supplement: S11 Fig — Metal binding residues differentiating Fe and Mn SOD activity marked with red boxes. Putative FeSOD accession numbers: Asterionellopsis glacialis, MMETSP1394, [CAMPEP:0197135456]; Odontella sinensis, MMETSP0160, [CAMERA:0183305568]; Extubocellulus spinifer, MMETSP0696, [CAMERA:0178491238]; Ditylum brightwellii, MMETSP1062, [CAMERA:0180922322]; Chaetoceros cf, MMETSP1336, [CAMERA:0119456406]; Skeletonema costatum, MMETSP0013, [CAMERA:0113409648]; Thalassiosira oceanica, [GenBank:EJK73388]; Thalassiosira pseudonana, [JGI:263062]; Thalassiosira punctigera, MMETSP1067, [CAMERA:0172528302]; Detonula confervacea, MMETSP1058, [CAMERA:0172326082]; Stephanopyxis turris, MMETSP0794, [CAMPEP:0195508046]. Putative MnSODs accession numbers: Amphora coffeaeformis, MMETSP0316, [CAMERA:0170658046]; Phaeodactylum tricornutum, [JGI:42832]; Nitzschia sp, MMETSP0014, [CAMERA:0113451442]; Fragilariopsis kerguelensis, MMETSP0735, [CAMERA:0170940508]; Pseudo-nitzschia arenysensis, MMETSP0329, [CAMERA:0116142848]; Asterionellopsis glacialis, MMETSP1394, [CAMPEP:0197142074]; Thalassionema frauenfeldii, MMETSP0786, [CAMERA:0178920282]; Nanofrustulum sp, MMETSP1361, [CAMPEP:0202481740]; Odontella aurita, MMETSP0015, [CAMERA:0113546944]; Minutocellus polymorphus, MMETSP1070, [CAMERA:0181044600]; Ditylum brightwellii, MMETSP1062, [CAMERA:0180939824]; Skeletonema costatum, MMETSP0013, [CAMERA:0113403410]; Cyclotella meneghiniana, MMETSP1057, [CAMERA:0172267766]; Detonula confervacea, MMETSP1058, [CAMERA:0172309720]; Chaetoceros sp, MMETSP0200, [CAMERA:0176485652]; Stephanopyxis turris, MMETSP0794, [CAMPEP:0195522388]; Rhizosolenia setigera, MMETSP0789, [CAMERA:0178949314]; Leptocylindrus CCMP1586, MMETSP1362, [CAMPEP:0196811398]. (TIF) [file pone.0129081.s011.tif]

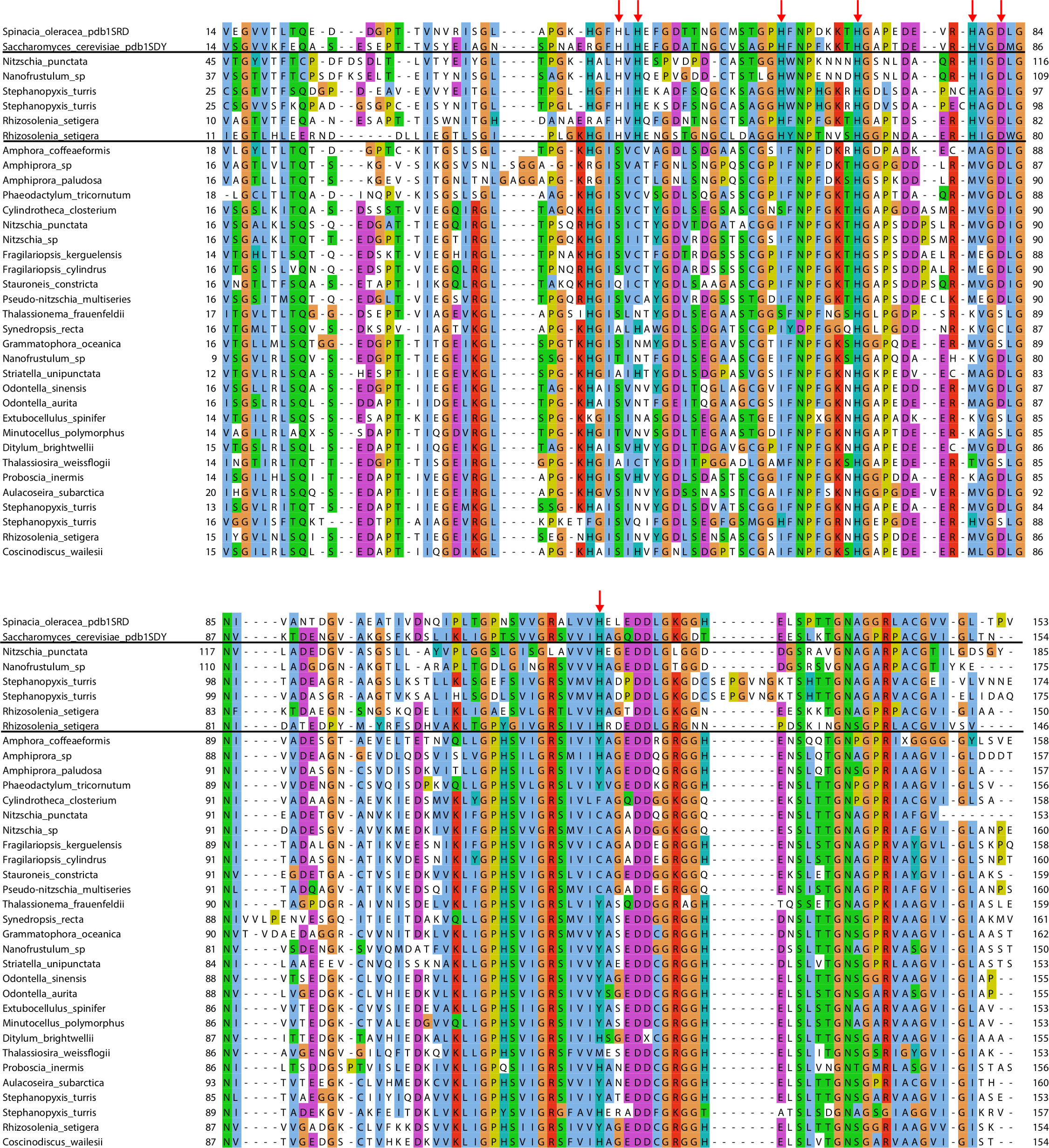

Supplement: S12 Fig — N-terminus sequence trimmed to aligned region at position 14 of S. oleracea. Reference sequences (top), diatom sequences that maintain all binding residues (middle) and other diatom homologs (bottom), separated by horizontal black lines. Key Cu and Zn binding residues marked with red arrows. MMETSP and accession IDs: Spinacia oleracea, [PDB:1SRD]; Saccharomyces cerevisiae, [PDB:1SDY]; Rhizosolenia setigera, MMETSP0789, [CAMERA:0178950748]; Stephanopyxis turris, MMETSP0794, [CAMPEP:0195542640]; Stephanopyxis turris, MMETSP0794, [CAMPEP:0195519432]; Nitzschia punctata, MMETSP0747, [CAMERA:0178859982]; Nanofrustulum sp, MMETSP1361, [CAMPEP:0202480120]; Rhizosolenia setigera, MMETSP0789, [CAMERA:0178941642]; Stephanopyxis turris, MMETSP0794, [CAMPEP:0195524364]; Thalassiosira weissflogii, MMETSP0879, [CAMERA:0171359706]; Thalassionema frauenfeldii, MMETSP0786, [CAMERA:0178894792]; Grammatophora oceanica, MMETSP0009, [CAMPEP:0194033848]; Synedropsis recta, MMETSP1176, [CAMERA:0119013068]; Amphiprora sp, MMETSP0724, [CAMERA:0168741714]; Amphiprora paludosa, MMETSP1065, [CAMERA:0172471802]; Amphora coffeaeformis, MMETSP0316, [CAMERA:0170661724]; Phaeodactylum tricornutum, [JGI:15852]; Pseudo-nitzschia multiseries, [JGI:220645]; Fragilariopsis kerguelensis, MMETSP0735, [CAMERA:0170910884]; Fragilariopsis cylindrus [JGI:269494]; Nitzschia punctata, MMETSP0747, [CAMERA:0178882228]; Nitzschia sp, MMETSP0014, [CAMERA:0113522044]; Cylindrotheca closterium, MMETSP0017, [CAMERA:0113603408]; Stauroneis constricta, MMETSP1352, [CAMERA:0119562388]; Nanofrustulum sp, MMETSP1361, [CAMPEP:0202482534]; Striatella unipunctata, MMETSP0800, [CAMERA:0118702026]; Extubocellulus spinifer, MMETSP0698, [CAMERA:0178620380]; Minutocellus polymorphus, MMETSP1070, [CAMERA:0181043470]; Proboscia inermis, MMETSP0816, [CAMERA:0171322060]; Aulacoseira subarctica, MMETSP1064, [CAMERA:0172423284]; Rhizosolenia setigera, MMETSP0789, [CAMERA:0178948996]; Odontella sinensis, MMETSP0160, [CAMERA:0183307194]; [file pone.0129081.s012.tif]

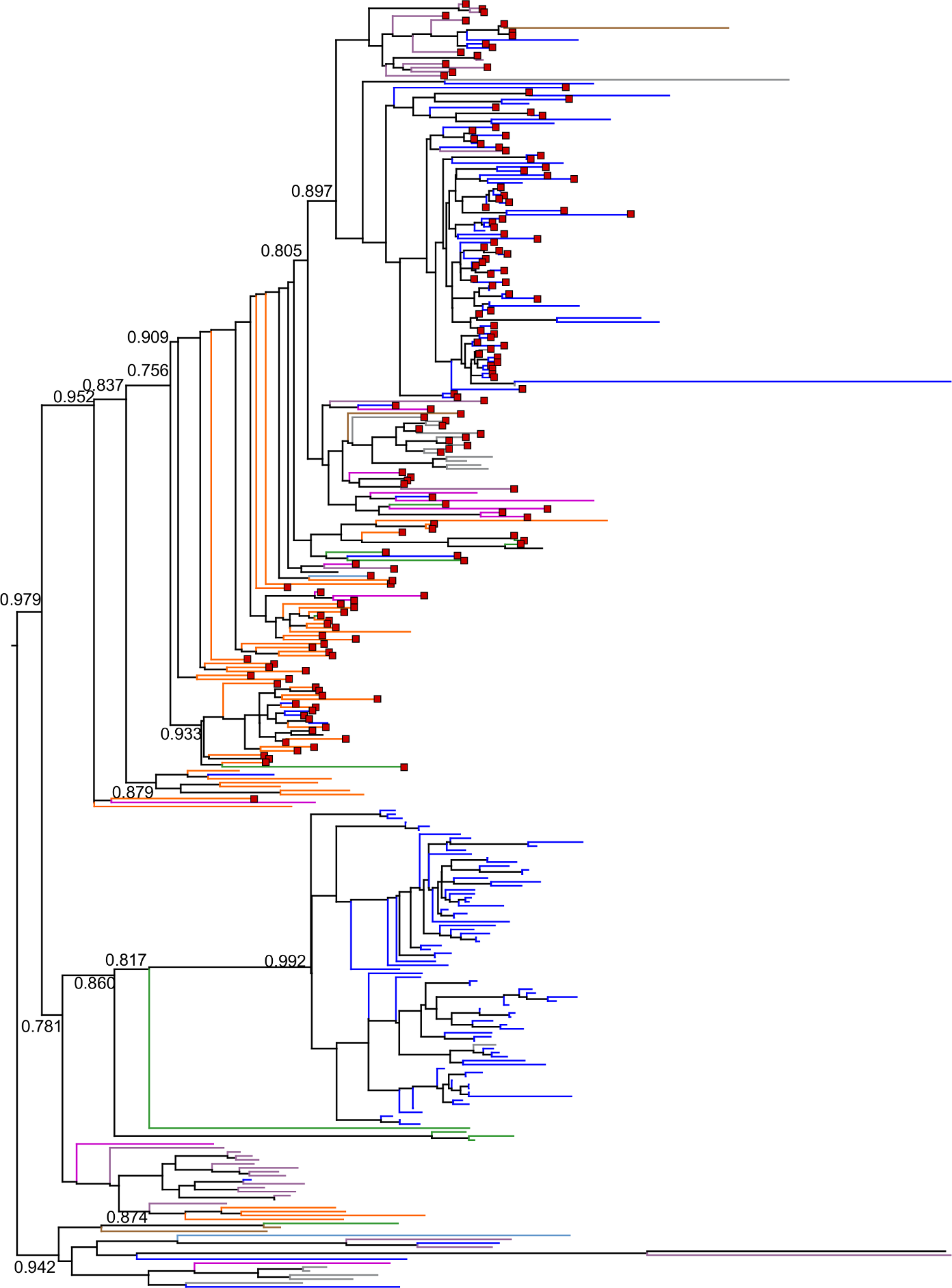

Supplement: S13 Fig — Midpoint-rooted approximately-maximum-likelihood tree of putative NiSOD amino acid sequences. Node support values are calculated from 1,000 resamples, only values over 0.5 are shown. One representative is shown from groups sharing greater than 95% similarity in aligned sequence identity. Sequences with an UBQ-coding region on the N-terminus are indicated by red squares. UBQ and other residues preceding the Ni-hook were trimmed from NiSOD-coding region prior to phylogenetic analyses to reflect relationship of NiSOD only. Branches colored by organismal phylogeny: diatoms, orange; chlorophytes, green; haptophytes and cryptophytes, lavender; non-diatom stramenopiles, magenta; alveolates, blue; unikonts, brown; excavates, pale blue; rhizaria, gray. (TIF) [file pone.0129081.s013.tif]
